# Main Ingredients for Success in L2 Academic Writing: Outlining, Drafting and Proofreading

**DOI:** 10.1371/journal.pone.0128309

**Published:** 2015-06-05

**Authors:** Rosa Munoz-Luna

**Affiliations:** Department of English, French and German, University of Málaga, Málaga, Spain; University of Westminster, UNITED KINGDOM

## Abstract

Spanish undergraduates of English Studies are required to submit their essays in academic English, a genre which most of them are not acquainted with. This paper aims to explore the extralinguistic side of second language (L2) academic writing, more specifically, the combination of metalinguistic items (e.g. transition and frame markers, among others) with students’ writing strategies when composing an academic text in L2 English. The research sample conveys a group of 200 Spanish undergraduates of English Studies; they are in their fourth year, so they are expected to be proficient in English academic writing but their written production quality varies considerably. Results are analysed following a mixed methodology by which metalinguistic items are statistically measured, and then contrasted with semi-structured interview results; SPSS and NVivo provide quantitative and qualitative outcomes, respectively. The analyses reveal that undergraduate students who produce complex sentences and more coherent texts employ a wider range of writing strategies both prior and while writing, being able to (un)consciously structure and design their texts more successfully. These high-scoring students make more proficient use of complex transition markers for coherence and frame markers for textual cohesion; their commonly used (pre-)writing strategies are drafting, outlining, and proofreading.

## Introduction and Research Objectives

The current situation in language teaching curricula in Spain is that foreign language learners do not feel capable of performing communicative strategies efficiently despite having studied a foreign language for years. University students of English Studies illustrate this point clearly as they are the utmost example of long-term L2 training and lack of L2 competence in both written and oral skills. As an experiential approach to this problem, I am tackling this issue from the point of view of L2 academic writing strategies, aiming to discover reasons for learners lack of proficiency beyond the evident linguistic inaccuracies. I believe that real L2 academic writing improvement requires an efficient use of certain strategies not all students are aware of or learn during their study process.

Writing has been always perceived as a field that is difficult to measure, assess, analyse and quantify [1–2]. Nowadays, it is still considered a very demanding activity, with many skills and sub-skills behind [3]. Due to its complex nature, the process of writing has been progressively linked to external issues that can possibly have an influence on it:

Writing expertise, as with literacy, cannot be removed from its historical and cultural contexts, and it cannot be described in terms of a naïve reduction to given cognitive procedures [4].

As a consequence, the study hypothesis leading the present piece of research is formulated as follows: explicit knowledge and active use of existing L2 writing strategies in the academic genre improves L2 undergraduate writing. According to this hypothesis, the primary objectives of our study are the following:

- To analyse undergraduates’ L2 written scripts from linguistic perspectives, measuring statistically certain discursive markers.
- To explore extra-linguistic factors that can influence undergraduates’ academic writing in L2 such as writing strategies prior to and during the writing process.
- To examine the relationship between students’ academic writing strategies and the quality of their written scripts in L2 English.

## The Strategic Nature of L2 Academic Writing

Writing is one of the most complex activities necessary for human literacy development. Therefore, it involves a series of actions related to curriculum and school instruction; this literacy element is present in the study and use of the written language by means of grammar and semantic instruction. Literacy dictionaries and handbooks on academic writing compile theoretical concepts on reading and writing. However, in order to draw the boundaries of writing, it has to be perceived first as a multidisciplinary and challenging activity, where many theories from varied nature converge [5–7].

Writing activities are an exercise of social relations where authors exchange ideas. Rather than focusing on the regularities of the academic writing style, I will look for writing idyosincracies in L2, that is, specific features that uncover writers’ involvement in what they are writing, as well as their awareness of contents and genre as they write. In this way, and to show the different extralinguistic factors affecting L2 academic writing, I am focusing on the different uses of writing strategies employed by university students when they write in L2 English, more specifically, those pre-writing strategies that help them plan their piece of writing.

The analysis of pre-writing strategies implies the need to explicitly teach those in the language classroom. In the case of L2 academic writing, students have the added issue of using a foreign language when writing. As this study results will show, the awareness and use of pre-writing strategies make a difference in the academic genre. If this academic strategic awareness is active, students’ texts will contain:

- Specific strategies consciously employed both in the text preparation and composition
- Signs to make the text available to the reader
- Better academic discourse organisation
- High scoring lexical and grammatical academic features

Teachers and readers cannot forget that these students’ writings are produced in a language that is not their own. They also have to bear in mind that writing is such a cognitive process that it is very similar no matter which language the writer is using. For this reason, students who lack first language strategies display a similar lack of strategies for writing in their second language [8]. Consequently, the teaching of academic writing goes beyond a list of syntactic and discursive uses of the written language only.

L2 academic writing is clearly composed by a set of layers of varied nature and purpose. All of them are important but writers cannot pay attention to all of them at the same time. Therefore, teachers have to plan writing strategies that students can consciously prepare and work on their texts from a multiple perspective, as strong L2 writers do; this multiple perspective includes (from [9]):

- Grammatical level: morphology and syntax, word and sentence formation
- Lexical level: lexicon and vocabulary; word register
- Discursive level: cohesion and coherence, transition between sentences and ideas
- Metadiscursive level: extralinguistic items and writers’ awareness of genre specifications
- Genre specifications: format, text structuring, target audience awareness, field
- Content compilation: text content according to topic and layout

## Extra-Linguistic Components of Undergraduates’ L2 Academic Writing: The Use of Academic Writing Strategies

Pioneering works on study strategies were those by [10–11]. The former already saw a need for students to learn to manipulate their own cognitive processes for academic purposes; O’Malley, et al. narrowed down the issue of students’ strategies to EFL learning. Research history of study skills in relation to EFL goes back to 1970, when some works were published regarding practice material for EFL students [12].

Students do not seem familiar with the different writing stages or strategies, and that is crucial to have good quality outcomes and to improve their L2 writing skills: writing strategies make them more autonomous and self-regulated in terms of written production in a foreign language. Writing strategies are necessary for writers to refine their ideas in their academic text production. As writing is a cyclical process, writers have to continually revise and change their scripts, and therefore change their writing strategies accordingly [13].

In opposition to the act of speaking, writing has been defined as a recursive process [14], this recursiveness being especially relevant in the context of academic written production. Recursive behaviour in academic writing ends up in a higher mark [15]: re-reading and drafting allow for a better written expression. The property of recursiveness is something to take into account in this work: as we will see in the quantitative script analysis, students who normally proof-read and revise their written pieces always tend to perform better.

Memory is, inevitably, a key aspect in the learning of a foreign language and it is an element to be taken into account in classroom activities. Although an eminently memoristic approach could be monotonous and decontextualised, learners need to memorise certain uses and concepts inevitably. Closely linked to mnemonic processes, we can find specific writing strategies such as the writing of key words to plan writing. Other cognitive and metacognitive strategies that complete the process of writing are:

- Re-reading and relating the text with other text parts
- Going back and forth in the writing process
- Using lexical analogies and discursive organization
- Summarizing
- Underlining, among others

Those additional techniques used at the time of writing for academic purposes can be defined as academic strategies. According to [16], students need more than linguistic knowledge in their academic career. In fact, that is what I saw in my script analysis: learners’ academic style does not generally present obtrusive mistakes; however, some of their written scripts seem much more academic than others. The use of strategies seems to be an important extralinguistic (non-linguistic) reason why this happens.

[17–18] shed some light on the way students perceive and use learning strategies in the field of EAP, as well as on their writing differences. The analysis of strategies in the process of L2 vocabulary acquisition has also revealed that foreign lexicon is better learned when learners focus strategically on form. Writing using target words also enhances this lexical learning [19], that is, form awareness is needed in order to improve certain aspects of L2 learning. However, issues on language awareness and strategy awareness have not been explicitly linked in research studies before; learners’ strategies have not been treated from an awareness perspective, but they have been merely described in relation to students’ results instead.

According to [20–22], the following learning strategies can be identified, which I have adapted to the field of L2 academic writing; all of them came up during students’ interviews, being therefore considered as crucial strategies for them (see Table 1).

**Table 1:** Writing strategies in L2 academic writing.

Writing strategies in L2 academic writing	
*Strategy*	*Description*
Metacognitive strategies	Planning, monitoring, reviewing, evaluating, reporting findings, recognising essay structures
Cognitive strategies	Repetition, organisation, summarising, imagery using, deducing, inference, note writing, paraphrasing
Comprehension strategies	Re-reading
Socio-affective strategies	Cooperative planning

In the light of the previous literature in the field of L2 academic writing and writing strategies, the present article aims to add a fuller perspective of L2 academic writing features and processes. Writers’ accounts of their own writing processes complete the academic genre picture by providing the most constructivist and inside part of writing.

## Methodology

When dealing with academic writing, mixed methods are a research option as these cover a wide range of circumstances co-occurring in these academic scenarios, namely: writers’ roles, writing processes and strategies, among others [23–25].

### Step 1: Discourse analysis for the study of L2 academic writing strategies

The script sample for discursive analysis conveys 200 essays that were written from 2000 until 2011(preliminary study, N = 30 written scripts; pilot study, N = 70 written scripts; final study, N = 100 written scripts), all of them produced by English Studies Spanish native undergraduates. Every script has been analysed following nine linguistic categories divided into three language levels (see Table 2).

**Table 2:** List of items to be examined in each script.

**A. LEXICAL LEVEL**		
**Language Item**	**Item Code**	**Description**
Code glosses	CG	Cohesion markers (e.g. for example, for instance, namely)
Lack of attitude or subjectivity markers	SUB	Attitudinal expression (agree, disagree, correctly, fortunately)
Lack of Spanish use	L1	L1 visibility in examples or explanatory notes
**B. GRAMMATICAL LEVEL**		
**Language Item**	**Item Code**	**Description**
Self-Mention	*SELF*	Self-mention in pronouns
Word order	*WO*	SVO order in sentences
Complex sentences	*COM*	Use of complex sentences and subordinators
**C. DISCURSIVE LEVEL**		
**Language Item**	**Item Code**	**Description**
Transition markers	*TM*	Coherence markers (accordingly, additionally, although, therefore, in contrast)
Frame markers	*FM*	Sequencing, stage labelling (firstly, in conclusion, in this section)
Punctuation	*PUNC*	Use of punctuation

Quantitative variables in the study.

Students’ L2 texts (which are naturally-occurring language samples) were analysed and coded according to the linguistic categories described below. Item analyses were based on whether they were present in the text or not, and we also counted up the number of instances of each item. After that, all scripts were ranked according to the number of features they had. The list of items to be examined (Table 2) includes language elements [26–28, 24, 29], ranging from single words (lexical level) up to segments or sentences (grammatical and discursive levels).

These markers have been counted up and analysed statistically with measures of central tendency using SPSS 16. In order to avoid out layers, median and mode have been also calculated; these values indicate the sample distribution regarding their results, as well as the tendency subjects show towards certain values.

### Step 2: Semi-structured interviews for the analysis of academic writing strategies

A qualitative stage was added in order to cover personal factors that equally affect students’ academic writing strategies. Those participants selected for the semi-structured interviews followed a process of random case sampling; interviewees were always chosen among written scripts authors. Interview questions were adapted from [30–32]. Our semi-structured interview questions follow [33] schedule for interviewing (see S1 Appendix).

1. Questions 1, 2 and 3 dealt with subjects’ attitudes about the academic world and writing.
2. Questions 4–8 deal with students’ behaviour and reactions when facing an academic text in English. Some questions were indirect since indirect techniques, also called projective techniques [33], help to reveal respondents’ most natural and spontaneous answers (e.g. question 5 from S1 Appendix).
3. Questions 9–16 refer to academic writing strategies and script quality.

When analysing interview results, we made category measurement by means of NVivo coding: demographic data were classified into cases and examined by means of attitude scales and attributes. After that, we made an axiomatic or crossed analysis in matrices, which related the different observations. Another stage within the coding process was the creation of coding profiles. Some patterning emerged from the mass of collected data as different writing behaviours appeared together with writers’ academic profiles. The direct consequence of this prototype design is the development of theories which explain L2 academic writing by undergraduates. These theories have been elaborated by means of NVivo reports and models.

### Study subjects

The study subjects are 200 undergraduates taking Linguistics modules as part of their English Studies degree programme. Participants in this study share the following features:

- They belong to a stratified random sampling [34] to justify our sample selection within the group of undergraduate students of English Studies at a Spanish southern university.
- They are in their third and/or fourth year (22 years old on average).
- None of them is a native speaker/writer of English.

The students’ written scripts used in our discourse analysis are part of their course formative evaluation, all written samples sharing the same formal features: (1) Students have to write short essay-like compositions; (2) Participants have two hours to write their essays, covering issues on theoretical and applied linguistics.

As this piece of research involved human participants, the study was reviewed and approved by the University of Málaga Ethics Committee before the study began, being Mr. Miguel Porras the Head of the Board. Prior to the collection of both written data and interviews, participants gave their written informed consent to the author of this paper. Students’ written consents are stored together with their written data at the Department of English, French and German Philologies in the University of Málaga. The Ethics Committee aforementioned approved this consent procedure.

## Results

### Quantitative results: written script analysis with SPSS 16

Table 3 illustrates the main descriptive statistical figures from a univariate perspective (analysing Table 2 quantitative variables separately).

**Table 3:** Descriptive statistics in the written scripts.

	N	Range	Minimum	Maximum	Means		Typ. dev.
CG	100	22	0	22	5,00	,444	4,440
NO_SUB	100	1	0	1	,49	,050	,502
NO_L1	100	1	0	1	,23	,042	,423
SELF	100	47	0	47	12,42	1,038	10,384
WO	100	55	0	55	23,77	1,373	13,729
COM	100	50	0	50	13,18	1,132	11,318
TM	100	32	0	32	8,65	,719	7,189
FM	100	17	0	17	1,84	,326	3,256
PUNC	100	194	22	216	95,93	3,798	37,976
MARK	100	9	1	10	5,42	,206	2,060
N	100						

Correlation among these nine variables is measured by means of Pearson’s correlation; this correlation analysis is lineal and it is used to describe both the type and intensity of the relationship between two variables. In this case, Pearson’s correlations show that the most relevant relations between variables are those taking place between any variable and the discursive ones. This fact implies that there is a linear relationship between grammar/lexical knowledge and discursive content in the text, which increases significantly as the writer is more skilled in grammar and lexical issues. Therefore, discursive competence seems to be key to make a script better in terms of academic features. Students discursive strategies when writing academically will also prove crucial regarding marking.

According to the study results, the presence of metalinguistic items is directly proportional to the academic mark each script receives. As Table 4 shows, we can find high correlations (over 0.7) between six of these study variables; in other cases, medium correlation (0.3–0.6) suggests that they are still indicators of academic success. *PUNC*, *TM*, and *COM* (i.e. punctuation, transition markers and complex sentences) maintain strong correlations with the final script mark, while *SELF* and *WO* are the variables with the lowest correlation. In other words, *COM* and the discursive level do make better written scripts, but the use of personal pronouns and English word order do not seem to affect L2 writing quality significantly.

**Table 4:** Inter-element relationship matrix.

	CG	NO_SUB	NO_L1	SELF	WO	COM	TM	FM	PUNC	MARK
CG	1,000									
NO_SUB	,281**	1,000								
NO_L1	,328**	,463**	1,000							
SELF	,299**	-,038	-,027	1,000						
WO	,403**	-,011	,108	,325**	1,000					
COM	,576**	,418**	,498**	,199*	,615**	1,000				
TM	,557**	,501**	,485**	,205*	,321**	,773**	1,000			
FM	,446**	,295**	,445**	,273**	,270**	,637**	,624**	1,000		
PUNC	,545**	,391**	,446**	,183	,490**	,767**	,704**	,539**	1,000	
MARK	,485**	,473**	,519**	,195	,393**	,753**	,756**	,574**	,778**	1,000

* Relevant inter-element relationship

** Highly relevant inter-element relationship

Consequently, academic writing strategies should be directed towards the development and improvement of complex sentences and discursive markers mainly. Once inter-element correlations were established, we carried out a multiple lineal regression, where the *y* dependent variable is what we want to predict, namely, student’s success in academic writing. We carried out a forwards stepwise regression model to see which of the 9 metalinguistic variables are actually relevant in predicting academic writing success; the model turned out to be significant. The following formula accounts for its significance: *F* = 56.735; *R* ^2^ = 0.692; *p*<0.001;

Using ANOVA test, Sig. = 0 indicates that the null hypothesis is not valid, strengthening our own research hypothesis. Finally, the multicollinearity analysis ensures that these linguistic variables are highly correlated (VIF under 10, and tolerance over 0.1 provide a reliable model) (Table 5 and Table 6).

**Table 5:** Anova Test.

Model		Square sums	Gl	Quadratic Means	F	Sig. ^a^
1	Regression	296,031	4	74,008	56,735	,000
	Residual	123,921	95	1,304		
	Total	419,952	99			

^a^ Predicting variables: *COM*, *FM*, *PUNC*, *TM*.

**Table 6:** Multicollineality analysis.

Model		Autovalues	Condition index	(Constant)	*PUNC*	*FM*	*TM*	*COM*
1	1	4,112	1,000	,00	,00	,02	,01	,01
1	2	,568	2,690	,06	,01	,44	,00	,00
1	3	,190	4,648	,15	,00	,54	,18	,15
1	4	,095	6,567	,00	,01	,00	,79	,57
1	5	,035	10,875	,78	,98	,00	,02	,27

This writing model confirms that the four variables: *COM*, *FM*, *PUNC* and *TM* are the most important predictors for academic writing success in Spanish undergraduate writers. Those scripts containing a higher amount of complex sentences, punctuation marks and adverbs receive higher marks than others. These writers employ a wider range of L2 academic genre writing strategies (as shown in interview results below) and are able to provide longer and more complex academic texts, combining content and form successfully; their scripts include:

1. Complex sentences in L2 (grammatical level).
2. Frame markers to divide the written discourse into argumentative segments, e.g. introduction, enumerations, discussion, conclusions (discursive level).
3. Correct punctuation (discursive level).
4. Transition markers to make the written discourse coherent (discursive level).

Once I obtained these data, I carried out a backward stepwise lineal regression in order to obtain a simplified model to predict academic marks in L2 writing:

$$\hat{y}=2.072+0.022\cdot PUNC+0.092\cdot TM+0.036\cdot COM$$

The previous formula indicates that low-scoring authors do not take risks when writing: their sentences are simple and basic, using diagrams and enumerations instead of *FM* and *TM*, therefore making their scripts more schematic. In some cases, these low-scorers present an excessive use of *SELF*. There are L1 transfers (interferences) at both grammatical and discursive levels, consequently triggering a certain phenomenon in the acquisition of L2 writing proficiency: when grammatical and lexical stages are poor, discourse is even poorer, and the opposite happens in strong writers (i.e. grammatical and lexical stages are stronger, and so their discursive connectors are more meaningful and complex too. See S1 Deidentified Essay 1 and S2 Deidentified Essay). In low-scoring authors, adverbs are of contrast only, and sentences, though simple (i.e. containing one verb only), can be eight-line long.

The following scripts belong to the lowest part of the continuum, where students’ writings present less (meta)discursive features; some of these cases are illustrated here with real writing instances:

“This led linguistics to called data and applying scientific methods in the investigations” (Script 04-2009-H)

“It is rationalist, and studies the languages from diachronically” (Script 04-2009-H)

“Strategies we take to create a coherent comunication due to performance variable or incompetence” (Script 04-2009-H)

“The use of recordings to observate certain characteristics is used, how they interact and/or influence each other, and so on” (Script 05-2009-M)

“The firsty thing you have is a language problem use, hypothesis, then, you collect data by tools, after that, you analyze the data and to finish you interpret the results” (26-2009-H)

The previous examples present mistakes on a lexical and vocabulary level; however, even if their lexicon is right, their adverbs (*TM* and *FM*) introduce lineal concatenations of events, producing simpler sentences and poorer descriptions. Stronger writers are able to compose complex sentences with subordinated verbs, while weak ones are limited to one-verb phrases. Moreover, high-scoring students structure their production in clearer paragraphing and in a sequential order: e.g. definition, typology, description, explanation, and conclusion. The following scripts belong to the highest part of the continuum, where students’ writings present more (meta)discursive features; these cases are also illustrated with real writing instances below:

“Eventually we find Pragmatics, which arose in 1980s with Applied Linguistics. it focuses on the study of language put into practice, that is, they prefer to study semantics, rather than just sentence structures” (02-2009-H)

“Humboldt was an empiricist, as opposed to his predecessors, the Port Royal Grammarians, who were rationalist” (13-2009-H)

“Traditional Linguistics is the first paradigm, and it is prescriptive which means that it tries to establish a set of norms and rules by which language should work” (13-2009-H)

As we can see, stronger writers are able to compose complex sentences while weak ones do not write long phrases. For the latter, it seems they are not ready to do so, or they do not feel ready to use English so freely and creatively. As [35] indicate, students’ self-concept is what ultimately motivates them to achieve a better performance. Only in skilled writers is there a clear structural layout in their written scripts: high-scoring students structure their production in clearer paragraphing and in a sequential order, and they are able to do so maybe because they know they can. For this reason, texts are more than cognitive procedures: they are key instruments of communication between the reader and the writer; texts mirror writers’ academic behaviour and knowledge of the academic genre.

These results are in line with previous research findings [36] stating that metacognitive strategies are linked to reflective behaviours in more autonomous learners. Moreover, better L2 academic writing results in a more coherent written expression. According to [37–38], that is an indicator of a well-developed constructivist process, where coherence means an optimum achievement of new understandings. Those scripts presenting a higher number of cognitive and metacognitive elements include a wider range of specific purposes in their discourse, that is, explanations, exemplification, descriptions, counter-arguments, claims, etc.

### Qualitative results: semi-structured interview analysis with NVivo 8

This qualitative data analysis starts during the actual interview process. The thirty semi-structured interviews were analysed using NVivo 8. The interviewees were the weakest and the strongest writers according to my results from the previous quantitative discourse analysis carried out before. This NVivo software allows for a full description and coding of the dialogues, while it establishes correlations among the different participants. The aim with this coding system is to have a list of categories that develop from the data and, at the same time, contain the main ideas.

Once all interviews were coded into different categories, I created free nodes to see the most relevant emergent topics that participants talked about. With all my resources coded, I drew tree nodes which helped to categorise the previous free nodes into topics and subtopics, making the whole analysis easier. The next step was to design a casebook where all participants and their main features were included, and also general matrices which served as overviews to see the topics dealt with in the interviews. The main topics which arose in the interviews coincide with the three external variables used in this study. In addition, students expressed their views on what good academic writing meant for them. A clear division emerged between those who highlighted the importance of coherence and cohesion (i.e. strong writers), and those who did not (i.e. weak ones).

The first variable matrix crossed two variables of the study: L2 writing strategies and participants’ awareness of the academic genre. In this way, we can see the relations established between each subject and the type of writing strategy they used when writing academically. Results show that there is a strong connection between proofreading while writing and a correct identification of academic genre instances; moreover, those participants who proofread while they write consider formal expression as important as contents when writing, something that enhances their script quality. Furthermore, those who show more autonomous strategies use outlines and drafts; these subjects identify textual genres correctly.

We can see again how strong writers present more positive and relaxed attitudes towards writing and towards their own university degree; although they can perceive some difficulty in the field of academic writing, they are able to overcome those problems and they identify textual genres correctly. Finally, there is a striking difference in the way they revise and proofread their scripts: high-scoring writers reread while they write, while weak writers do not revise or they do it once they have finished writing; in this way, strong writers are able to modify their writings as they write, improving their final outcomes and having better academic results. Low reading skills end up in poor comprehension and even low self-esteem. In this case, students who do not proofread their scripts present poorer results than the others. On the other hand, skilful readers use a wider range of academic vocabulary and resources; they can select meaning more easily while language is being processed in their minds.

## Discussion of Results: The Influence of (Pre)Writing Strategies in L2 Academic Writing Quality

When analysing written scripts in relation to students’ interview statements, some key ideas emerge that provide the basis for my data analysis. For this reason, my results discussion is based on a cross-case analysis: common aspects in students’ responses help to develop the different themes discussed. Regarding my variable of academic writing strategies, [12] analyses some writing techniques which are also mentioned by students in their accounts of their academic writing: summarising, proof-reading, drafting and organising their time. In this study, I can make a further division in these strategies by grouping them into two categories: firstly, those related to memory processes, and on the other hand, those dealing with a deeper reading comprehension, outlining and planning.

Regarding the use of writing strategies in L2, subjects can be divided into clear-cut groups depending on their strategic use of language and techniques when they are writing academically. Those participants who are not successful in self-regulating activities, do not focus on personal progress (i.e. internal cognitive processes), but rather on external indicators: grades, peer comparisons, etc. On the other hand, self-regulation and self-confidence in academic writing seem to be crucial in the production of quality scripts. According to the results obtained in this paper, I have divided the previous students’ writing strategies into the following categories:

- Surface strategies: students do not change their internal text structures. They primarily focus on word choice; normally used by less-skilled writers.
- Deep strategies—metastrategies [22]: text planning, which can be divided into:
  - Advanced planning: by skilled writers, who plan their scripts before they start to write.
  - Emergent planning: by less-skilled writers, who design their text form and content as they write.
- Transformational strategies: text changes. These strategies allow writers to rewrite sentences and change text focus, both in content and form.

Each of the aforementioned categories will correspond to a specific L2 writer profile, depending on their writing abilities: skilled writers, for instance, spend more time on planning both content and discourse before actually writing, and they also make transformational changes more easily. The use of these strategies has a clear influence not only in the text production process but also in the final result quality.

According to these results, those students using memory strategies produce poorer scripts and show less awareness of the academic genre (cf. 30.D.♀ and 05.D.♂). Moreover, they admit they never proofread their essays before submitting them, and their drafts are mainly for mnemotechnic purposes, writing down some words just not to forget them: “when I read, I try to memorise some expressions which I think are going to be useful in writing, and then I memorise them” (30.D.♀). On the contrary, 10.F.♂ and 11.F.♀ are able to describe a full range of writing strategies related to planning, outlining ideas and proof-reading, which helped them to use academic features consciously from a deeper understanding: “I always make an outline, or a draft, as a help or support to what I want to write later. That helps you to write sequentially” (11.F.♀).

The use of strategies and prior planning in the production of an academic essay is generally considered as a crucial step in the composition of any academic text. In the same way, proofreading becomes not only a useful textual practice but also something that makes a difference between good and bad written scripts. Both text planning and revision are something that strong academic writers are aware of as our interview results show. As [39] argue, language planning and structure seem to precede meaning in language processing; when extraposing these results to language processing stages, it seems that structure comes first when writing as well.

If we combine their use and awareness of writing strategies with their scripts, we can see the role of metadiscourse in academic writing. A notion and mastery in metadiscursive features help students with the construction and revision of their own written production [16]. In fact, writers who present a higher number of metadiscursive items produce better quality papers and make use of more writing strategies in their writing process. As far as genre awareness is concerned, strong writers are able to give a full account on academic genre definition and features. On the other hand, weak writers fail in giving a definition of the academic genre, though they have received the same formal instruction about genre as the strong ones in their degree.

The strong writers taking part in this research process can be described as self-regulating learners. Better writers are metacognitive, motivated and strategic. In fact, if we correlate their scripts with their interviews, we can see that they show a greater awareness of metadiscursive features, a more positive attitude, and a wider range of writing strategies.

Strong academic writers construct a writer persona or identity; this implies the use of explicit metadiscursive and language resources mentioned in our analysis. Skilled-writers seem not to translate from their mother tongue: the proof is that those scripts containing more L1 influence are poorer and receive lower marks. Therefore, translation from/into L1 is possibly a technique to use at beginner levels but not with proficient writers. L1 transfer is a useful resource for weaker writers, but “counter-productive” in stronger ones.

Academic writing in English is particularly difficult for Spanish undergraduates since there is a long cultural and philosophical tradition behind it. [40] have identified some differences between continental writing and Anglo-Saxon writing in academic text types:

- Continental tradition is more philosophical, interpretative, epistemological and digressive
- Anglo-Saxon writing is linear, empirical, and to the point

Taking these differences into account, students’ grammatical mistakes seem to be minor details compared to text internal structuring. However, this continental vs. Anglo-Saxon dichotomy is not being examined in this article (what could be part of future research projects, using the data collected for this one).

With regard to the relationship between text type and text production, some work has shown that genre-based strategies instruction improves the ability to produce effective tokens of that genre. However, there is little reference to how learners analyse the targeted genres before actually engaging in a writing task, and this being especially relevant in the field of ESAP. As we can deduce from the results of this study, there are some crucial differences between L1 and L2 writers that affect their final written products. L2 writers in particular tend to:

- Write their ideas straight away without previous planning
- Have difficulties in setting writing goals and create new material
- Proofread without any reflection on their texts

We can therefore divide the L2 writing process into three steps, which are increasing in complexity:

- Search for linguistic accuracy
- Search for creativity and originality, manipulating ideas and making use of more complex discursive rules
- Metacognitive stage, when students are conscious of their own mistakes and try to correct/avoid them

Likewise, there is a clear strategy in the production of meaningful academic texts:

- Strategic writing plan
- Action and set of actions
- Writing profile
- Payment of feedback: the comments the writer receives once their writing has been read, or the expected use of their text

## Conclusions

The multiple perspectives and disciplines underlying the study of L2 academic writing just sheds some light on the complex process and nature of such field. By dividing the writing process into quantitative and qualitative aspects, we are aiming to provide a more comprehensive insight of academic texts, which follow both individual intuitions and pre-established collective formats. This group of Spanish undergraduates builds their texts upon coherence and cohesive principles, while they also make use of extralinguistic strategies and academic genre awareness to produce their scripts. Therefore, in order to have a fuller view of academic textual production, we have carried out discourse analysis and semi-structured interviews to gather metalinguistic elements and (pre)writing strategies, respectively. SPSS and NVivo 8 have served both these purposes.

As a result of textual codification and interview analysis, we can state that strong writers make use of a wider variety of metalinguistic items in their academic texts: their scripts are not only cohesive but also coherent. As far as their extralinguistic behaviour is concerned, skilled writers enjoy writing in a foreign language, and their writing task awareness involves textual comprehension and deep proofreading while they are producing a text.

On the contrary, weak writers do not show academic features awareness and their texts are simpler and shorter: simple lexicon, simple sentences, and no risks taken when writing (See S3 Deidentified Essay and S4 Deidentified Essay, respectively). They perceive academic writing as a difficult and demotivating task, and they hardly ever proofread what they write. As a consequence of their lack of proofreading, they do not change what they write (i.e. no rewriting or error correction). Less-skilled writers make use of superficial strategies, paying attention to word morphological aspects and not to paragraphs and coherence matters. The contrast between both writing behaviours results in academic mark variations.

The importance of writing strategies and genre features awareness are easily forgotten by foreign language practitioners. However, the advantages of teaching ESAP genre awareness go from grammatical issues to even identity theories. Students will not only obtain better marks but they will be learning and reflecting upon attitude awareness, target-reader needs, etc., which would improve their L2 writing practice. These results go in line with Toussaint and Clarks’ (2008), who found the need for social variables and personal relationships in the learning of a second language. When designing a syllabus for ESAP, we should dismiss the creation of an overly artificial academic language graded for students of English as a foreign language; on the contrary, we should follow a more pragmatic and multi-modal criterion where students’ real needs and motivations are the target.

## Supporting Information

S1 Deidentified Essay 1 (PDF)

S2 Deidentified Essay 2 (PDF)

S3 Deidentified Essay 3 (PDF)

S4 Deidentified Essay 4 (PDF)

S1 Appendix (DOCX)
