# Supplementary material for: Main Ingredients for Success in L2 Academic Writing: Outlining, Drafting and Proofreading
Source: PLoS One. 2015 Jun 5;10(6):e0128309. doi: 10.1371/journal.pone.0128309 (PMC4457904; doi:10.1371/journal.pone.0128309)
Supplement: S1 Deidentified Essay 1 — (PDF) [file pone.0128309.s001.pdf]

Primer Parcial — 8
Segundo Parcial — 9 > 8'5
SOBR.

12, 2009, d

LINGÜÍSTICA INGLESA: CORRIENTES SINTÁCTICA ACTUALES

Depto. Filología Inglesa, Francesa y Alemana (Universidad de Málaga)

NAME AND SURNAMES: [redacted]

ID NUMBER: [redacted] **DATE:** 19 / 06 / 2009

FIRST TERM

1. Do only verbs assign theta roles? Provide some examples to illustrate your opinion. NO

2. What can you tell me about the Pro-drop parameters? sujetos

3. Analyse the following example: is it (un)grammatical? Comment on the role of *there* in the sentence:

There occurred three accidents after lunch. logical subject / sintactic subject.

SECOND TERM

1. Analyse the following sentences (remember that the use of tree diagrams is voluntary):

a) Poirot preferred to be an excellent teacher rather than a brilliant doctor.
6.1 b) Poirot turned out to be an excellent teacher rather than a brilliant doctor.
6.2 c) Poirot was liable to be an excellent teacher rather than a brilliant doctor.

2. Are the following sentences (un)grammatical? Explain why:

a) I consider very much him to be a good candidate. YES
b) Miss Marple surely gave her pipe to Janvier. NO

3. What is exceptional about this sentence?

For him to have agreed to the proposal is surprising.

4. Try to rescue these examples using what you know about Case Theory and/or c-command domains:

a) *Your parents to come to my wedding would be a smart move.
b) *Mary's concern him.
c) *Poirot travelled John and me.
d) *Patrick$_1$ should wash themselves$_1$ every day.

5. Look at the following sentence: "My grandmother believed my boyfriend to be a liar":

a) Is the sentence grammatical or ungrammatical? Why?
b) Now look at the second part of the sentence: [my boyfriend to be a liar]: is it a CP or an IP? Why?

6. Analyse the following sentence using as much theoretical support as you can:

He was fascinated by everything.

UNIVERSIDAD DE MALAGA
DEPARTAMENTO DE FILOLOGIA INGLESA, FRANCESA Y ALEMANA

Alumno D.
Centro
Asignatura
Curso
Fecha

FIRST TERM

3/3

(1) Do only Esta página entera . roles? Provides ~~some~~ examples to ~~provide~~ ill von.

No, verbs are .. only ones to assign theta roles. ~~although~~ for example, he buys him a car. In this case, the verb can assign three different arguments, although "him" can be omitted, for example he buy a car. Then, we put this verb like this. ~~be~~ buy → [NP] ([NP]) [NP]. We have three arguments but one of them can be omitted.

According to the theta criteria, each argument is only assigned a theta role and each theta role is only assigned to an argument. Then, "he" will be assigned a theta role, in this case "AGENT", and "a car" is the theme of sentence.

But, we can see in the following examples as an adjective and a preposition can assign a theta-roles, for example, in "Jane is envious of me"; In this example, the copula verb "to be" is weak and almost transparent to assign a theta role. For this reason, ~~the~~ 'envious' assign a theta role to "Jane" and "of me" because we have two arguments of envious: [NP] envious [PP].

So, if we do not want to break the theta grid, we must

assign a theta-role to each argument.

At the same way it happens with the preposition "between", for example, in Genova is between Roma and Milan.
We have the same copular verb 'to be', which is weak and transparent in order to assign a theta-role. ~~But~~ so, the preposition "between" assign three arguments: [NP] between [NP][NP].

2/3

(2) what can you tell me about the ~~Prep~~ Pro-drop parameters?

the pro-drop parameters are related to Extended Projection Principle because it is related to the subject in each sentence.
SELF
As we know, the subject can be omitted in other languages, for example in the Spanish language: ~~[illegible]~~ Compré un coche.
TM
However, The English language cannot omit the subject because the inflection of english verbs are poor, for example I bought a car.
Lack of TM
But, there are some cases in which ~~a~~ subjects are presented in the sentence although they ~~were~~ not written, for example, in "To tell her ~~the~~ truth is not correct" and "I ~~consider~~ think to buy a new car.
In the first example, the subject of the clause does not appear, but we cannot break the Extended Projection Principle in which each sentence ~~subject~~ must ~~be~~ have a subject, so we put 'Pro$_{ARB}$' to indicate the place of subject, ~~a~~ as it is showed here:
TM-FM

Alumno D.

Centro

Asignatura

Curso

Fecha

SELF

PRO ARB shows us that we have a subject but we do not know exactly what is it. (ARB → arbitrary). we must add this subject should appear with the preposition for, [[for us / for you to tell the truth...] was...]
PRO ARB.

FM

In the other example, "I ~~consider~~ think to buy a new car", the subject of the clause does not appear but it is implicit, and we put 'PRO₁' to indicate it. PRO₁ indicates us that the subject is related to another part of the sentence, in this case "I". [I think [PRO₁ to buy a new car]].

End-weight?

3/4

③ Analyse the following example: is it (un)grammatical? Comment on the role of there in the sentence.

"there occurred three accidents after lunch."

Although this sentence can appear to be strange, it is perfectly grammatically, although there are some aspects we must note it.

SELF
TM

This sentence, for some linguists, has two subjects. One of them is the syntactic subject (there), and the other is

the logic one (three accidents). There is an insertion in the sentence and it is called ~~explet~~ expletive pronoun. This pronoun has not relevance to the meaning of the sentence and no role is assigned to it. So, we leave no role for there.

Impersonal ?
Pleonastic ?

UNIVERSIDAD DE MALAGA
DEPARTAMENTO DE FILOLOGIA INGLESA, FRANCESA Y ALEMANA

Alumno D. ....

Centro ....

Asignatura ....

Curso ....

Fecha ....

SECOND TERM

1/1

1a → It is a grammatical sentence because the verb "to be" is not repeated between "rather than" and "a brilliant doctor". we have a subordinate clause ("to be an excellent ... doctor"), in which the subject is implicit in the clause, as we see here "Poirot$_1$ prefered [PRO$_1$ to be an excellent teacher rather than a brilliant doctor]"

1/1

1b → Here we have a raising predicate from type one. As we know, we have a subject-to-subject movement in which Poirot moves from his initial position, between turned out and to be to his final position, at the beginning of the sentence. It equivalent?

1/1

1c → Here we have a raising predicate from type two. we also have a subject-to-subject movement in which Poirot moves from his initial position, between liable and to be, to his final position, at the beginning of the sentence.

1/1

2a → this sentence is ungrammatical. If we want this sentence was grammatical, we must insert the preposition "For" between very much and him. If we do this, there are nothing which blocks him in order to receive case, because the preposition would be the case-assigner for him.
the ~~[illegible]~~ verb to be cannot assign a case because it is a non-finite verb which it is not allowed to assign a case.
The other verb, consider, is not allowed to assign a case for him because it is blocked by very much.

1/1

2b → this sentence is grammatical because ~~the verb "give"~~ we find the three arguments of this verb (give), and because the adverb "surely" is a verb complement and it must appear close to the verb.

0'25/1

(3) this sentence is grammatical and it is exceptional because the heavy constituent, in this case a clause, is placed at the beginning of the sentence. We do this in order to emphasize the clause. *
it is also a peculiar sentence because of the insertion of the preposition "For" to avoid the ungrammaticality of the sentence. If we do not insert this preposition, the inflection is too weak to give a case.

ECM?

* A normal construction of this sentence would be [illegible]

UNIVERSIDAD DE MALAGA
DEPARTAMENTO DE FILOLOGIA INGLESA,
FRANCESA Y ALEMANA

Alumno D.

Centro

Asignatura

Curso

Fecha

him to have agreed the proposal. We would insert the dummy it and the heavy constituent would be placed at the end of the sentence.

0'5/0'5

4a → The inflection is too weak to assign a case so for this reason, we must insert the preposition "for" in order to make this sentence grammatical. 'for' percolates through the inflection and it ~~assigns~~ gives a case for 'your parents'.

0'5/0'5

4b → The noun phrase "Mary's concern" c-commands "him" and we know that a noun phrase cannot assign case. it is ~~necessary~~ necessary to insert something between the two noun phrases. In this case, the insertion of the preposition "about" would solve the ungrammaticality of the sentence.

0'5/0'5

4c → the verb "travel" requires a ~~#~~ noun phrase ~~as~~ and a prepositional phrase, so you cannot put Poirot travelled John and me. You must insert the preposition "with" in order to recover the grammaticality of the sentence.

0'25/0'5

(4d) → According to the Binding theory, the reciprocal pronoun themselves will be related to a plural subject. with more than one participant. In this case, Patrick is alone, so themselves cannot refer to Patrick. In order to solve this, we put "himself" in order to "themselves". c-command domain?

1/1

(5a) → this sentence is perfectly grammatical because there is no obstacle between the verb believe and the noun phrase "my boyfriend", so the verb is the case-assigner of the noun phrase.

(5b) → You can represent this clause as you want because "my boyfriend to be a liar" is the same than "that my boyfriend is a liar". So we can represent with a CP or an IP. In the CP we have the complementizer "that" and in the IP we have "to".

1/1

(6) - he was fascinated by everything. -

this is a passive sentence. the main features of the passive sentences are the following:

- the morphology of the verb changes.
- the elements of the sentence change its position, so the case is different in the passive voice than in the active voice, but the roles does NOT change "he" will be the "experiencer" in the active or passive voice

UNIVERSIDAD DE MALAGA
DEPARTAMENTO DE FILOLOGIA INGLESA,
FRANCESA Y ALEMANA

Alumno D.
Centro
Asignatura
Curso
Fecha

- the nominative case-assigner changes in the passive voice.
- the inherent case dissapear on the passive voice.

— [illegible] el final
