# Supplementary material for: Main Ingredients for Success in L2 Academic Writing: Outlining, Drafting and Proofreading
Source: PLoS One. 2015 Jun 5;10(6):e0128309. doi: 10.1371/journal.pone.0128309 (PMC4457904; doi:10.1371/journal.pone.0128309)
Supplement: S2 Deidentified Essay 2 — (PDF) [file pone.0128309.s002.pdf]

[redacted]

6 - 2009, d

Primer parcial — 7'5
Segundo parcial — 6'45 } 6'9
APR.

LINGÜÍSTICA INGLESA: CORRIENTES SINTÁCTICA ACTUALES

Depto. Filología Inglesa, Francesa y Alemana (Universidad de Málaga)

NAME AND SURNAMES: [redacted]

ID NUMBER: [redacted] **DATE:** 19.06.08

FIRST TERM

1. Do only verbs assign theta roles? Provide some examples to illustrate your opinion.

2. What can you tell me about the Pro-drop parameters?

3. Analyse the following example: is it (un)grammatical? Comment on the role of *there* in the sentence:

There occurred three accidents after lunch.

SECOND TERM

1. Analyse the following sentences (remember that the use of tree diagrams is voluntary):

a) Poirot preferred to be an excellent teacher rather than a brilliant doctor.
b) Poirot turned out to be an excellent teacher rather than a brilliant doctor.
c) Poirot was liable to be an excellent teacher rather than a brilliant doctor.

2. Are the following sentences (un)grammatical? Explain why:

a) * I consider very much him to be a good candidate.
b) Miss Marple surely gave her pipe to Janvier. ✓

3. What is exceptional about this sentence?

For him to have agreed to the proposal is surprising.

4. Try to rescue these examples using what you know about Case Theory and/or c-command domains:

a) *Your parents to come to my wedding would be a smart move.
b) *Mary's concern him.
c) *Poirot travelled John and me.
d) *Patrick$_1$ should wash themselves$_1$ every day.

5. Look at the following sentence: "My grandmother believed my boyfriend to be a liar":

a) Is the sentence grammatical or ungrammatical? Why?
b) Now look at the second part of the sentence: [my boyfriend to be a liar]: is it a CP or an IP? Why?

6. Analyse the following sentence using as much theoretical support as you can:

He was fascinated by everything.

UNIVERSIDAD DE MALAGA
DEPARTAMENTO DE FILOLOGIA INGLESA,
FRANCESA Y ALEMANA

Alumno D.

Centro

Asignatura

Curso

Fecha

FIRST TERM 2'5/3

(1) In a first impression people can think that only verbs can assign theta role but with the following proofs we can observe how other words have the same capacity. L1

(FM) Firstly, nouns assign theta role. For example (CG)

"Maria's analysis".

Here analysis gives to Maria the theta role of

Secondly, prepositions assign theta role too, an example of that are: "It is between the table and the chair" / "It was done by her". In the first example the preposition between assigns case to the table and the chair; the same happens with the second example where by assigns the role of agent.

Thirdly, adjectives also gives case to other words.

CG For instance:

2/3

(2) The PRO-DROP parameter makes reference to an omission of a pronoun. This pro is not the empty category of a deep structure, it makes reference to an omitted pronoun.

If we want to speak about that pro-drop we have to mention the principle of economy, because

that principle has the same objective: to omit words in order to say more without the use of them. This principle of economy and the pro-drop can appear only in languages that admit it because its grammaticall is not afected. For example in Spanish we can say "Llueve" but if we want to say that in English we have to say: "It is raining" because English follows the rules of Subject, Verb Object (SVO) and if we do not put the verb the sentence is ungrammatical. So, that pro-drop has to be related with languages that have a lot of inflexions, in their vocabulary. Other example is "They are crazy". Here we need a context to know if "they" means in spanish for example "Ellos o ellas". In Spanish we say directly "Ellas están locas".

?

3/4

(3) There, ocurred [three accidents], after lunch.

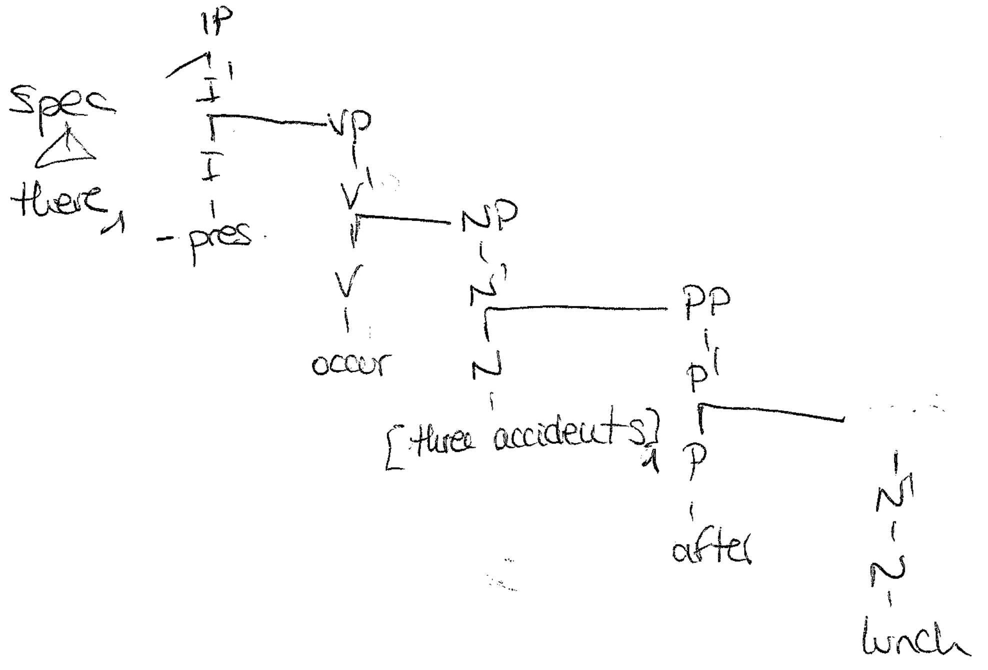

UNIVERSIDAD DE MALAGA
DEPARTAMENTO DE FILOLOGIA INGLESA,
FRANCESA Y ALEMANA

Alumno D.
Centro
Asignatura
Curso
Fecha

We have a grammatical sentence because it does not violate the projection principle. Firstly we have to mention that there and three accidents are co-index, in other CG words, they are refered to the same word, in this case to the three accidents. then when we know that we observe that we have the subject. Secondly FM each words has its theta role. The main inflection occurred assigns the role of agent (subject) to the three accidents and the preposition after gives case to lunch.

L1 It has to be mentioned also that the transitive verb "occur" has the needed Direct object.

the most destacable thing here is the "there" that appears at the beginning of the sentence. we have to say about it that it is here to emphasize the sentence. It is one of the types of "there" linked with the first characteristic that is when it appears for necesity, for CG example "there are three books" and the second one, which appears when we do not SELF have a concrete number of things: there are some books".

Type of there?

SECOND TERM : 0'5 / 1

① Poirot$_1$ prefered [PRO] to be an excellent teacher rather than a brilliant doctor.

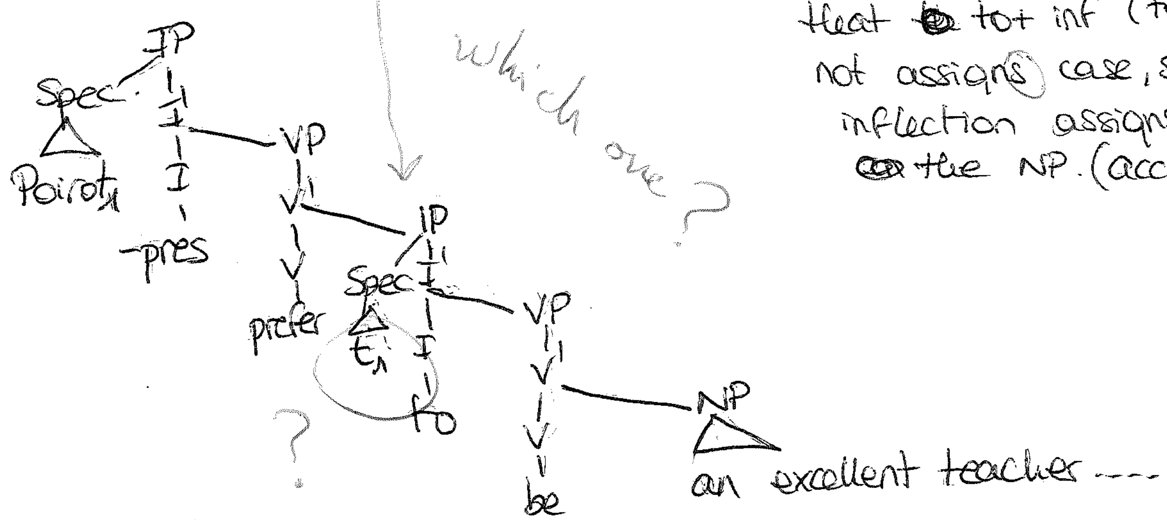

⊛ Here we have to say too that ~~the~~ tot inf (to be) does not assigns case, so the main inflection assigns case to ~~on~~ the NP. (accusative).

This is a grammatical sentence. The main inflection assigns nominative case to Poirot (Subject). As we see in ~~SELF~~ FM the diagram after the VP (prefer) we have a second IP but in this ocassion we do not have subject as in the first one, for this reason we need to use the empty category PRO to fill-in it, because if we do not use ~~the~~ it we violate the case filter due to the subject position would be empty. This pro tells us that in that position "supossly" is Poirot in the deep structure.

After that, there is not *bad expression in English* more problems because each word has its case and there is not any violation of the case filter. There are three characteristics that the case-assigners have in this sentence to justify all that I say:

- ~~The~~ A head cannot give case himself.
- The case assigner is followed by the word that it gives case.

[illegible]

UNIVERSIDAD DE MALAGA
DEPARTAMENTO DE FILOLOGIA INGLESA,
FRANCESA Y ALEMANA

Alumno D.

Centro

Asignatura

Curso

Fecha

0'3/1

(It) Poirot turned out to be an excellent teacher than an a brilliant doctor.

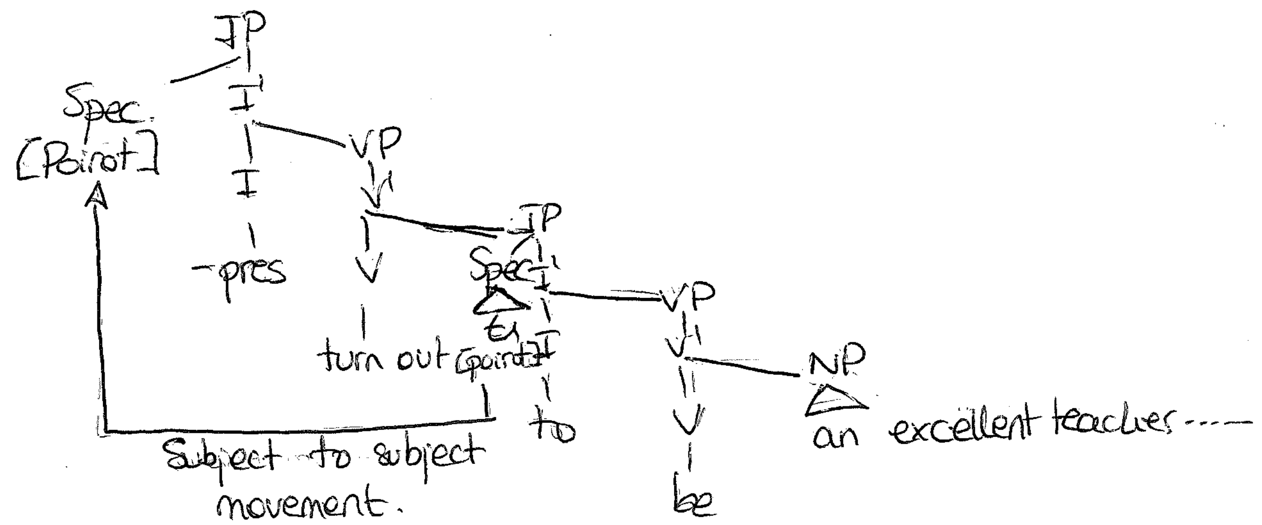

This sentence is grammatical too and the difference with the first one that it consist in a Raising Predicate. that verb belongs to the ~~third type of raising verbs called semi-auxiliaries.~~ ~~there~~ ~~to~~ prove that it is a real raising predicate we can substitute the subject (Poirot) for dummy it (It) and the sentence is also grammatical.

The most destacable in that raising predicate is the subject to subject movement because if we do not that we violate the projection priciple of the sentence. It consist into move the subject of the second inflection position to the first one, creating a trace in that place that indicates us it is refeared to the Spec → Poirot.

AS in the first sentence to+infinitive does not assigns case because it is ~~not~~ possible. The main inflection gives theta role to ~~the~~ NP.

ב'ר/1

Poirot$_i$ was liable PRO to be an excellent teacher rather than a brilliant doctor.

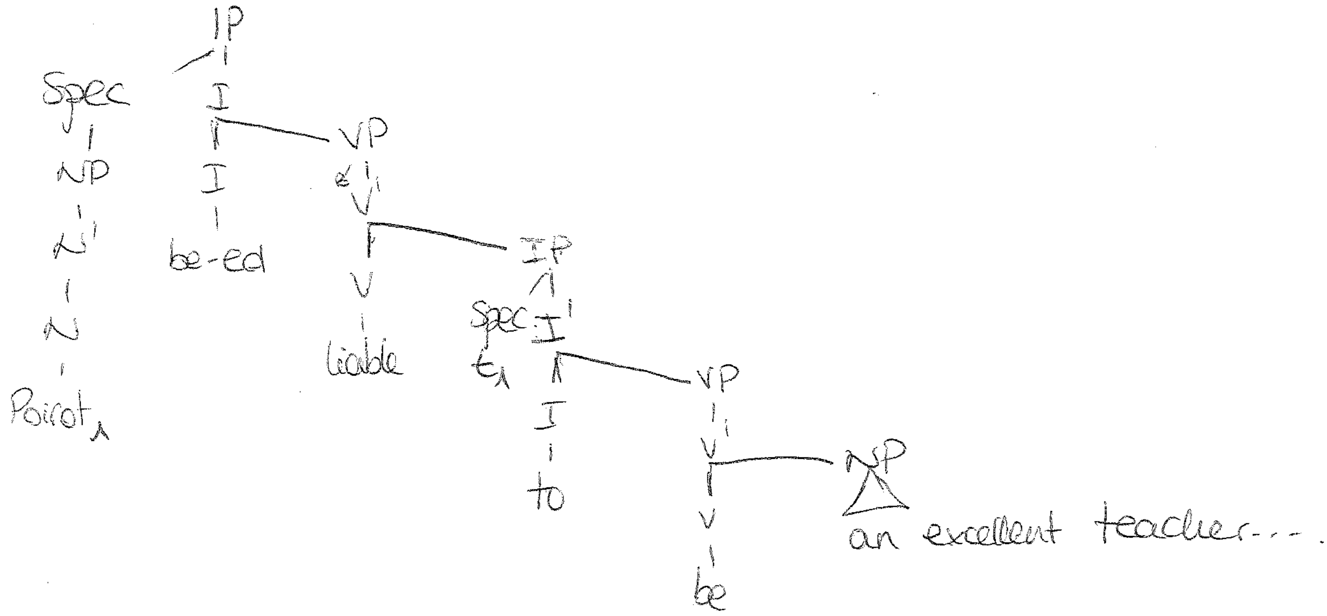

This is a grammatical sentence. The IP gives inflection to Poirot which is the subject and after that IP, we have a second IP (to), with the difference that here we do not have subject, so we need an category, in this time an empty category to supply that carence and by this way do not violate the case filter by which, all the words need to have their theta-role.

Appart of that, the sentence do not have any other problem because we have a NP at the end with a CP that is grammatical.

Raising predicate?

UNIVERSIDAD DE MALAGA
DEPARTAMENTO DE FILOLOGIA INGLESA,
FRANCESA Y ALEMANA

Alumno D.
Centro
Asignatura
Curso
Fecha

(2) 0'7/1

a) This sentence is ungrammatical because it violates the case filter. Here the IP has to give case to him and the adverb "very much" blocks it. To rescue the sentence it is needed a preposition that assigns case to him, if we do not have it, this sentence is not grammatical. which preposition and where?

0'5/1

b) This sentence is grammatical because what we have between the Inflection and Miss Marple is an ~~adjective~~? and this type of words can appear here or at the end of the sentence. It does not block anything. This is the main difference between the previous sentence; adjectives? do not need prepositions or any other word to constitute a grammatical sentence, it does not violate the case filter and everything is all right.

1/1

(3) In that sentence we have an Exceptional Case Marking (ECM) because we have an accusative working as a nominative. This case is "him". This case is exceptional because if we put "he" instead of "her" the sentence would be ungrammatical, it is needed to use the accusative form to create a correct sentence.

(4) 0'5/0'5

a) For Your parents to come to my weeding would be a smart move.

To rescue that sentence we have to insert the preposition for at the beginning of the sentence because if we do not put it there is any case assigner. Here the IP is to come but verbs to+infinitive cannot assign case, so for rescues the sentence because it gaves case to your parents. By this way we do not violate the case filter and the sentence is grammatical.

0'5/0'5

b) Mary's concern about him.

In my opinion to rescue that sentence we can introduce the preposition "about" because here we do not have a verb who assigns case to Mary's concern, so concern assigns case to Mary's but there is no element that assigns case to him and by this way we violate the case filter. then if we use about between concern and him, the preposition will give case to him and the sentence will be grammatic

0'5/0'5

c) Poirot travelled John and me.

Here we have the same situation as in the previous sentence. We violate the case filter and we need something to avoid it. For this reason I think that if we put the preposition "with" after the verb "travelled", the NP "John and me" will have case.

UNIVERSIDAD DE MALAGA
DEPARTAMENTO DE FILOLOGIA INGLESA, FRANCESA Y ALEMANA

Alumno D.

Centro

Asignatura

Curso

Fecha

0'5/0'5

d) Patrick, should wash ~~themselves~~ himself, every day

FM Here the situation is different than in the previous sentences. We know that Patrick governs themselves, so they have to speak about the same (in this case Patrick). So if Patrick's C-command domain is "should was themselves every day" we have to change themselves and put himself. By that way there is agreement between both words, because they are co-index.

(5) My grandmother believed my boyfriend to be a liar

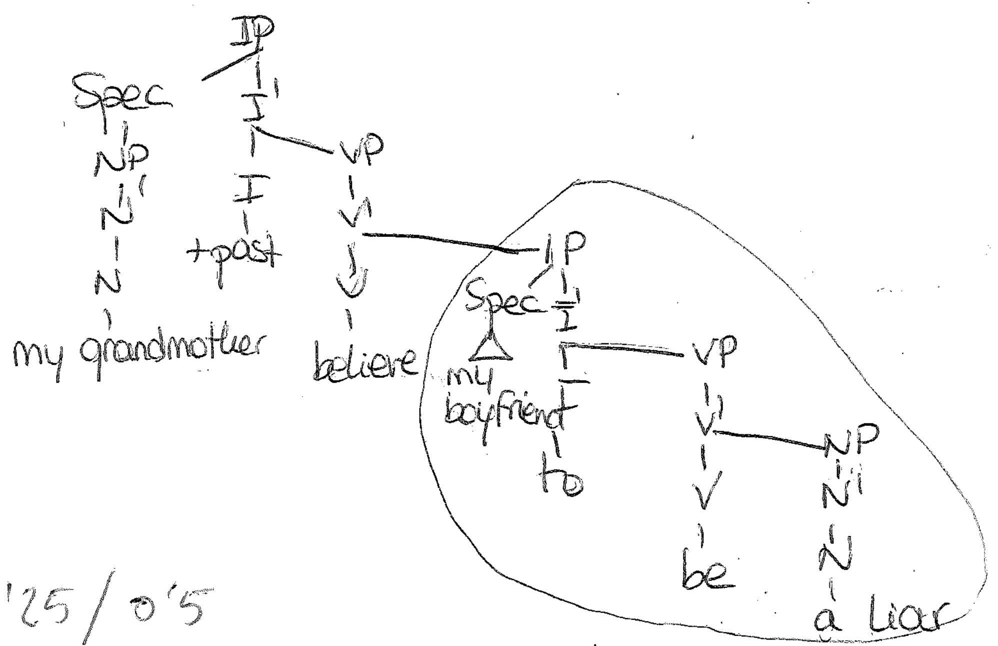

0'25/0'5

a) This sentence is grammatical because there is no violation of the case filter, each words have! their own case. ~~and~~ ~~to~~ In conclussion, there is anything wrong here as we can see in the previous tree.

expression/ ?

0/ 0'5

b) I ~~think it is an IP because as we can see in the previous diagram.~~

6. AGENT He was fascinated by everything PATIENT 1/1

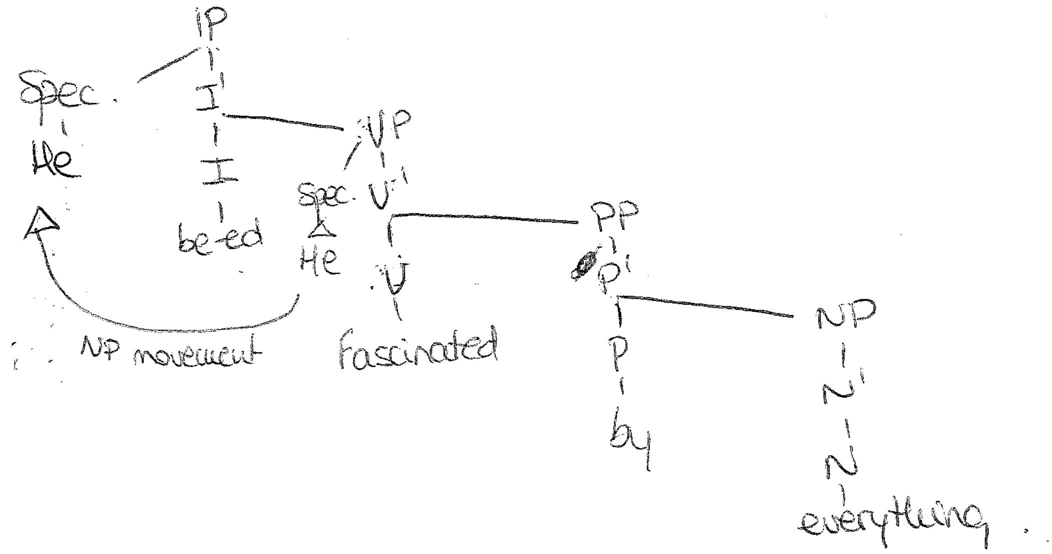

It is a grammatical sentence. As we can see there is no violation here. The IP gives nominative case to He (subject) and it is the agent of the sentence. This is a passive sentence as we observe because we have BE + PAST PARTICIPLE and a clear patient introduced by "by".

The inflection is 'was' because in the passivisation the auxiliary absorbs the ability of assign case that the main verb has = CASE ABSORTION.

Other characteristic of passives is that there is a noun phrase movement because if we do not have that, we violate the case filter, the subject position would be empty and the sentence would be ungrammatical.

And the last characteristic of pasivisation is that the agent is introduced by him. However this particle does not always appear; in sentences, it can be ommited if it obvious.
