# Supplementary material for: Main Ingredients for Success in L2 Academic Writing: Outlining, Drafting and Proofreading
Source: PLoS One. 2015 Jun 5;10(6):e0128309. doi: 10.1371/journal.pone.0128309 (PMC4457904; doi:10.1371/journal.pone.0128309)
Supplement: S3 Deidentified Essay 3 — (PDF) [file pone.0128309.s003.pdf]

Primer Parcial — 4'5
Segundo Parcial — 5'25 > 4'9
APROB.

5, 2009, d

LINGÜÍSTICA INGLESA: CORRIENTES SINTÁCTICA ACTUALES

Depto. Filología Inglesa, Francesa y Alemana (Universidad de Málaga)

NAME AN[redacted]

ID NUMBE[redacted]

FIRST TERM

1. Do only verbs assign theta roles? Provide some examples to illustrate your opinion.

2. What can you tell me about the Pro-drop parameters?

3. Analyse the following example: is it (un)grammatical? Comment on the role of *there* in the sentence:

There occurred three accidents after lunch.

SECOND TERM

1. Analyse the following sentences (remember that the use of tree diagrams is voluntary):

a) Poirot preferred to be an excellent teacher rather than a brilliant doctor.
b) Poirot turned out to be an excellent teacher rather than a brilliant doctor.
c) Poirot was liable to be an excellent teacher rather than a brilliant doctor.

2. Are the following sentences (un)grammatical? Explain why:

a) I consider very much him to be a good candidate.
b) Miss Marple surely gave her pipe to Janvier.

3. What is exceptional about this sentence?

For him to have agreed to the proposal is surprising.

4. Try to rescue these examples using what you know about Case Theory and/or c-command domains:

a) *Your parents to come to my wedding would be a smart move.
b) *Mary's concern him.
c) *Poirot travelled John and me.
d) *Patrick$_1$ should wash themselves$_1$ every day.

5. Look at the following sentence: "My grandmother believed my boyfriend to be a liar":

a) Is the sentence grammatical or ungrammatical? Why?
b) Now look at the second part of the sentence: [my boyfriend to be a liar]: is it a CP or an IP? Why?

6. Analyse the following sentence using as much theoretical support as you can:

He was fascinated by everything.

UNIVERSIDAD DE MALAGA
DEPARTAMENTO DE FILOLOGIA INGLESA,
FRANCESA Y ALEMANA

[redacted]

FIRST TERM

1'5/3

① Not only verbs can assign theta roles. [No FM, TH]

In the example: ~~[illegible]~~

He threw a ball to the window (the preposition 'to' [illegible]akes that 'the window' had the role of goal.
goal

In the example:

He came from Paris
↳ source

In the example:

The knife was in the box
↳ location

more assigners?

② Pro-drop parameters: 2/3 [SELF-WE]

English language does not allow ~~#~~ a subject pronoun [illegible]o be omitted. In Spanish, [we can] omit it when necessary, [illegible]ut in English [we] always have to have a noun or whatever [illegible]hich acts as a subject, otherwise, the sentence would [illegible]e ungrammatical. If [we are going] to write a ~~[illegible]~~ sentence [illegible]d we see that it ~~does~~ does not have a subject, we will have [illegible] insert a word which acts as a subject. For instance: [CG] [illegible]t insertion or there insertion:

English: It seems to be red. [NO]

EM !

As we have seen in the example before, in Spanish, it is not necessary to have ~~an~~ subject (it is implicit).

It is called in English 'expective there', when we use it to form the subject.

• Principle of economy: All these I have [SELF] been talking about is projected in this principle of economy: which says that Spanish language is much richer than English language so we can omit many words in Spanish, and the sentence will still have sense, it would be grammatical. Moreover, Spanish ~~[illegible]~~ language is reach in writing and oral skills, so it is a big difference from English.

In English we have a structure which is SVO and it has to be correctly 'done'. This is what projection principle says. In English, there have to be ~~an~~ subject + Verb + Object (complements are obligatory or not depending on the verb and context). NO TM

• Agreement and pro-drop.

In literature [WO] we can proove that agreement is very important

1/4

③ - There occurred three accidents after lunch -

It is a grammatical sentence. The word 'there' has been inserted to rescue the sentence and acts as the subject (Agent-θrole) → NO.

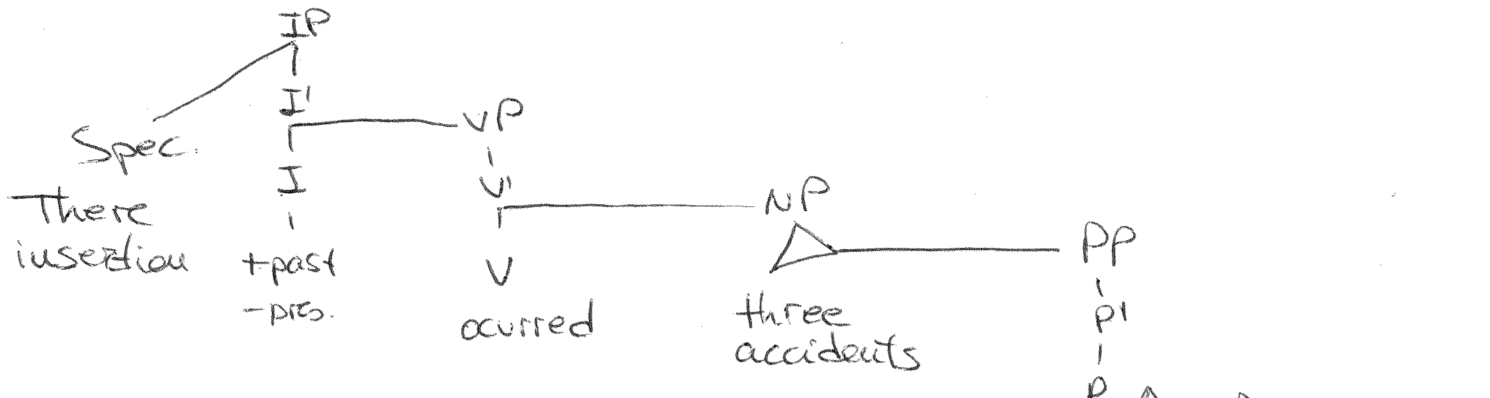

UNIVERSIDAD DE MALAGA
DEPARTAMENTO DE FILOLOGIA INGLESA,
FRANCESA Y ALEMANA

[redacted]

SECOND TERM

0'25/1

(1) a) Poirot preferred to be an excellent teacher rather than (PRO) a brilliant doctor.

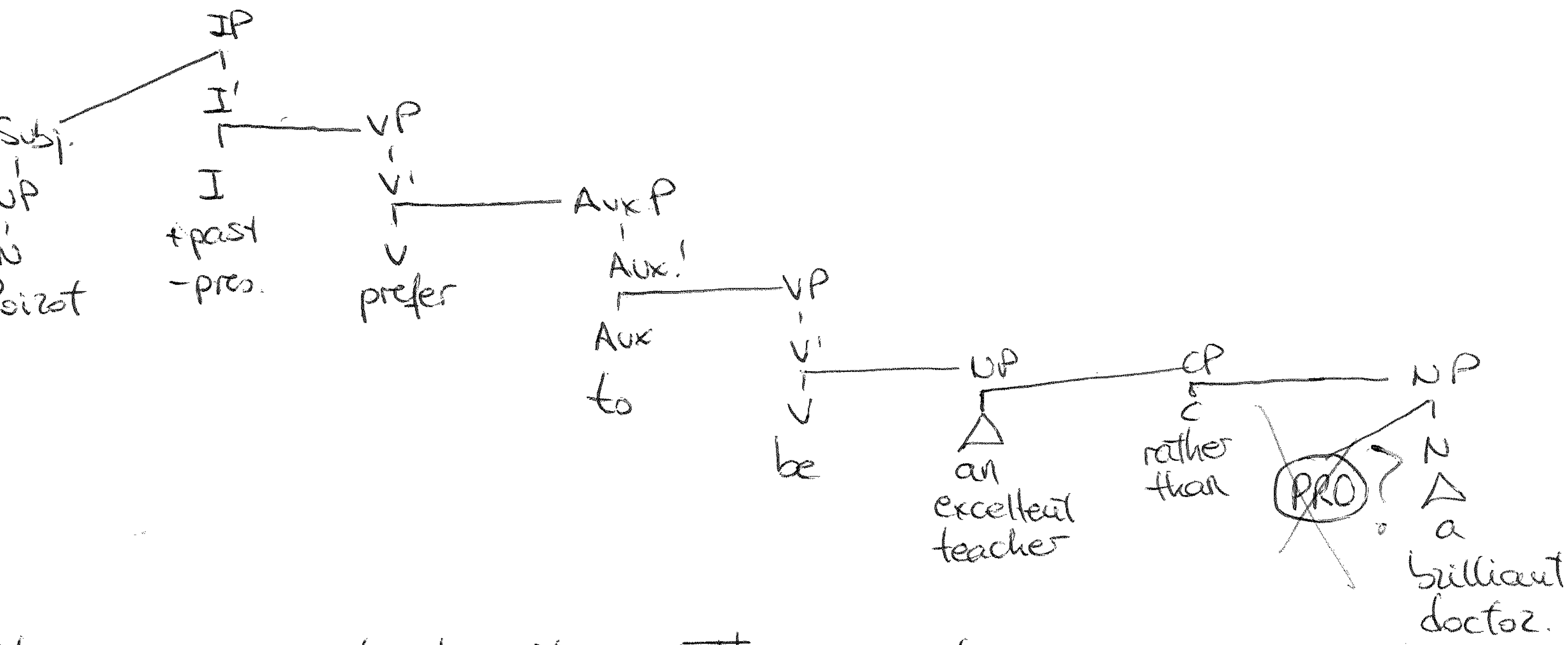

...t is a grammatical sentence. The projection principle and the ...se filter are not violated because it has a subject and every NP ...s a case. The part of the sentence 'to be an excellent teacher ...ther than a brilliant doctor' has the accusative case, assigned ... the verb (inflected verb) 'preferred'. There is a (PRO) between ...han' and 'a brilliant...', because otherwise, 'Poirot preferred ... be' would have to be repeated. There is a to+infinitive which ...es not assign case cause it can't. The PRO does not have ...ase neither.

The verb C-commands the rest of the sentence, ~~and governs~~.
↓
(prefer)

b) Poirot turned out to be an excellent teacher rather than (PRO) a brilliant doctor.

It is a grammatical sentence in which the projection principle is not violated (Poirot → Subject) and the case filter is assign (each NP has a case. It belongs to the ~~3rd~~ group of Raising Predicat[illegible] (verb → turn to)

The verb 'turned out' assigns accusative case to the rest. As in the previous example there is a (PRO) ?

FM

0'25/1

Subject-to-subject ?

c) Poirot was liable to be an excellent teacher rather than (PRO a brilliant doctor.

It is a grammatical sentence which belongs to the ~~3rd~~ 2nd typ[illegible] of raising predicates.

0'25/1

UNIVERSIDAD DE MALAGA
DEPARTAMENTO DE FILOLOGIA INGLESA,
FRANCESA Y ALEMANA

[redacted]

(2) Grammatical or ungrammatical?

1/1

a) I consider very much (him) to be a good candidate.

It is ungrammatical because the Case filter is violated. [illegible]n this example, 'him' does not have a case because 'much' and [illegible]e to + infinitive are not case assigners, so it is not correct [illegible]or grammatical). The verb 'consider' could assign case, [illegible]l there is something blocking it ('very much') → Adjacency.

1/1

b) Miss Marple surely gave her pipe to Janvier.

WO + FM

In this case, the sentence is grammatical because there [illegible] nothing blocking the case Filter. The verb 'gave' is the case [illegible]ssigner, and 'her pipe' would receive the case of DO.

case assigner.

0'8/1

(3) (For) him (to have agreed to the proposal) is surprising.

The clause between bracket would be the subject, because the concordance with the verb. 'For him' is a compl. *

In this case (exceptional case Marking), the sentence [illegible]egins with a preposition 'For him'. It is correct and [illegible]rammatical because 'for' (FM) can assign case so it resue [illegible]he sentence, otherwise, if we say: 'Him to have ...', [illegible] would be ungrammatical.

(4) *Your parents to come to my wedding would be a smart move.

For your parents, to come to my wedding, would be a smart,

If we introduce the preposition 'for', it would be gramma

0'5/0'5

*Mary's concern him.

Mary's what ~~concern~~ him.
CP

*Poirot travelled John and me.

X

*Patrick, should wash themselves, every day.

"I can't rescue it!" X

! Attitude.

UNIVERSIDAD DE MALAGA
DEPARTAMENTO DE FILOLOGIA INGLESA,
FRANCESA Y ALEMANA

[redacted]

0'7/1

(5) "My grandmother believed my boyfriend to be a liar".

a) It is grammatical because Projection Principle and [illegible]ase Filter are not violated. The inflected verb 'believed' is [illegible]e case assigner of 'my boyfriend'.

b) [my boyfriend to be a liar]

This is a CP. Why? FM Firstly, because the verb is [illegible]ot inflected (it is a to + infinitive). And secondly, because [illegible] we can say "My grandmother believed that my boyfriend [illegible]as a liar".

that → CP (complementizer)

(6) "He was fascinated by everything". 0'5/1

This is a grammatical sentence. It belongs to the 4th [illegible]pe of raising predicates (passive sentence)? The projection [illegible]inciple and the Case Filter are not violated, otherwise, the [illegible]ntence would be ungrammatical.

We can say that there is 'abstract case' because if we see [illegible]e pronoun 'He', we think that it is nominative in an active [illegible]ntence. We use 'he' because it is a passive. If we see the [illegible]eep structure, we can see all the movements in this kind [illegible] sentences: (arbolito detrás) L1!

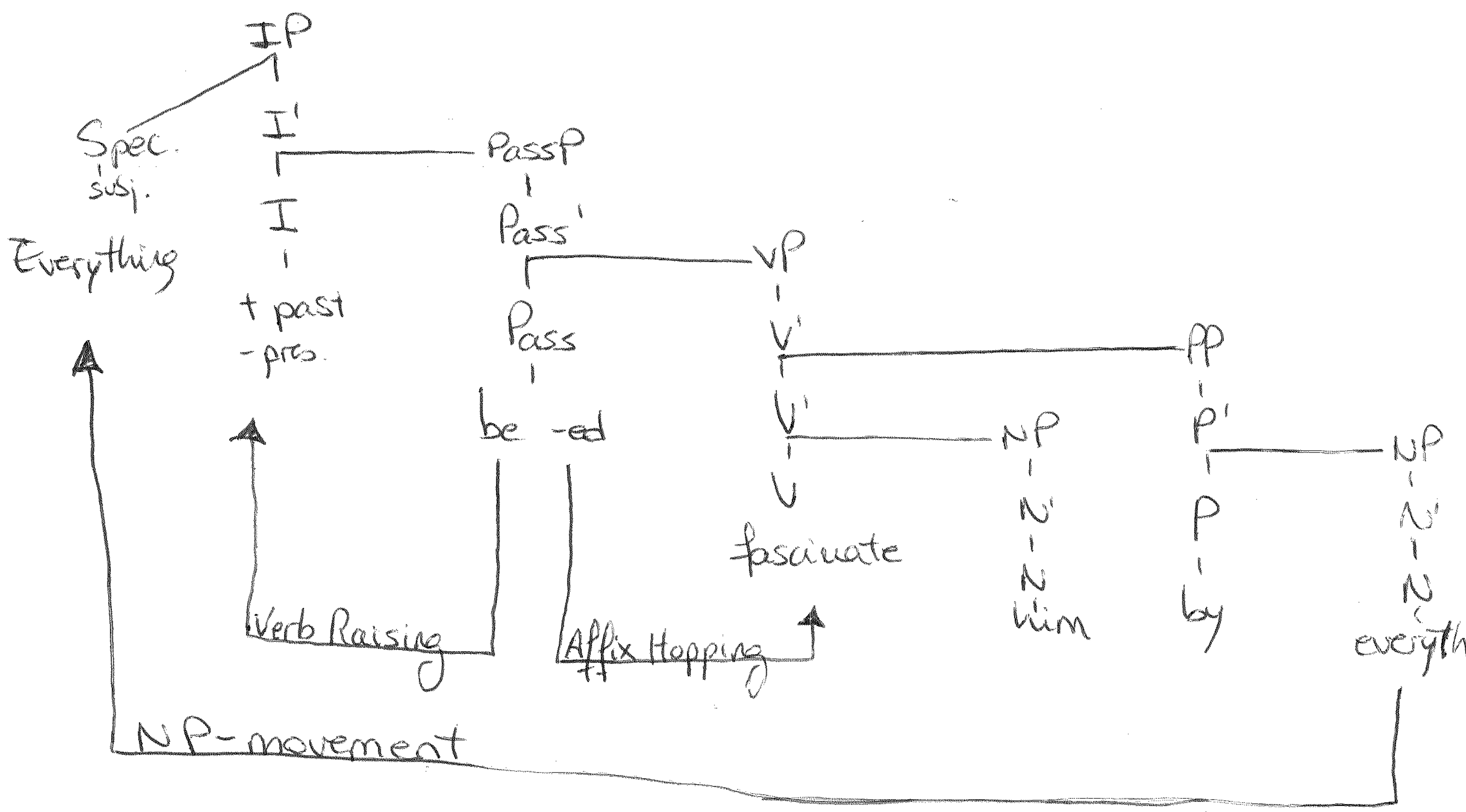

• The verb 'fascinate' C-commands 'him'. There is nothing blocking them (Adjacency), so it governs it as well.

* [At the same time, the verb C-commands the PP, but it does not govern it because there is a barrier blocking them (him)] → No porque el PP estaría ya situado en el Spe
