# Supplementary material for: Main Ingredients for Success in L2 Academic Writing: Outlining, Drafting and Proofreading
Source: PLoS One. 2015 Jun 5;10(6):e0128309. doi: 10.1371/journal.pone.0128309 (PMC4457904; doi:10.1371/journal.pone.0128309)
Supplement: S4 Deidentified Essay 4 — (PDF) [file pone.0128309.s004.pdf]

Primer Parcial — 1
Segundo Parcial — 1'9
SUSPENSO

8-2009, d

LINGÜÍSTICA INGLESA: CORRIENTES SINTÁCTICA ACTUALES

Depto. Filología Inglesa, Francesa y Alemana (Universidad de Málaga)

NAME AN[redacted]

ID NUMBE[redacted]

FIRST TERM

1. Do only verbs assign theta roles? Provide some examples to illustrate your opinion.

2. What can you tell me about the Pro-drop parameters?

3. Analyse the following example: is it (un)grammatical? Comment on the role of *there* in the sentence:

There occurred three accidents after lunch.

SECOND TERM

1. Analyse the following sentences (remember that the use of tree diagrams is voluntary):

a) Poirot preferred to be an excellent teacher rather than a brilliant doctor.
b) Poirot turned out to be an excellent teacher rather than a brilliant doctor.
c) Poirot was liable to be an excellent teacher rather than a brilliant doctor.

2. Are the following sentences (un)grammatical? Explain why:

a) I consider very much him to be a good candidate.
b) Miss Marple surely gave her pipe to Janvier.

3. What is exceptional about this sentence?

For him to have agreed to the proposal is surprising.

4. Try to rescue these examples using what you know about Case Theory and/or c-command domains:

a) *Your parents to come to my wedding would be a smart move.
b) *Mary's concern him.
c) *Poirot travelled John and me.
d) *Patrick$_1$ should wash themselves$_1$ every day.

5. Look at the following sentence: "My grandmother believed my boyfriend to be a liar":

a) Is the sentence grammatical or ungrammatical? Why?
b) Now look at the second part of the sentence: [my boyfriend to be a liar]: is it a CP or an IP? Why?

6. Analyse the following sentence using as much theoretical support as you can:

He was fascinated by everything.

UNIVERSIDAD DE MALAGA
DEPARTAMENTO DE FILOLOGIA INGLESA,
FRANCESA Y ALEMANA

[redacted]

- FIRST TERM: 1/3

1.) - Not only verbs assign a theta role; nouns, adjectives, prepositions and inflections also assign a theta role. L1

CG Ex.: - The boy is reading the book in the library.

explanation?

0/3

2.) - Pro-drop parameters are these ?

0/4

3.) – [There]Subj. (Spec.) [occurred] (V.) — Predicate: [three accidents] (Complement of quantity), [after lunch.] (Complement of time)

F.M (no relevant).

The use of there [in this case] is as a complement of the verb. It can be substituted by another element of the same category, because it is obligatory required by the verb. ?

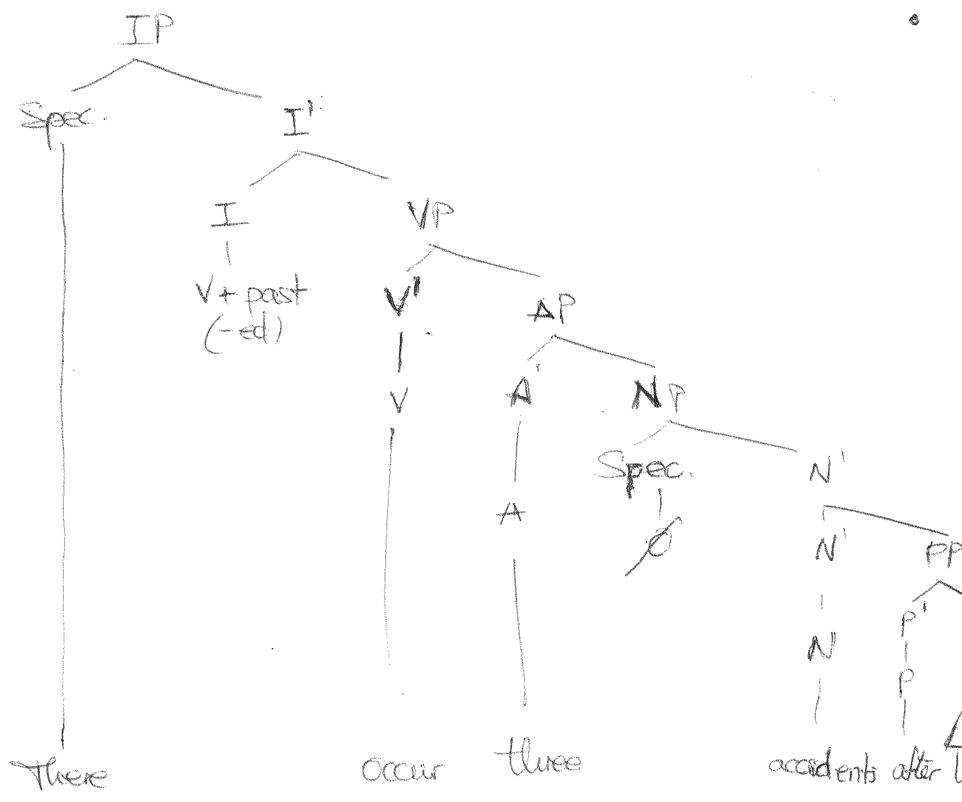

UNIVERSIDAD DE MALAGA
DEPARTAMENTO DE FILOLOGIA INGLESA,
FRANCESA Y ALEMANA

[redacted]

- SECOND TERM: 0'25/ 1

Prop. 1.
Pred.

1.) - a) - Poinot preferred to be an excellent teacher

Subj. V D.O. (Adv. clause)

Adv. of quality

Subj. 1, 2. (Nominal clause)

Prop. 2. Pred.

rather than a brilliant doctor. t_1

Superlative Adjunct.

Adv. of mode

D.O. (Adv. clause)

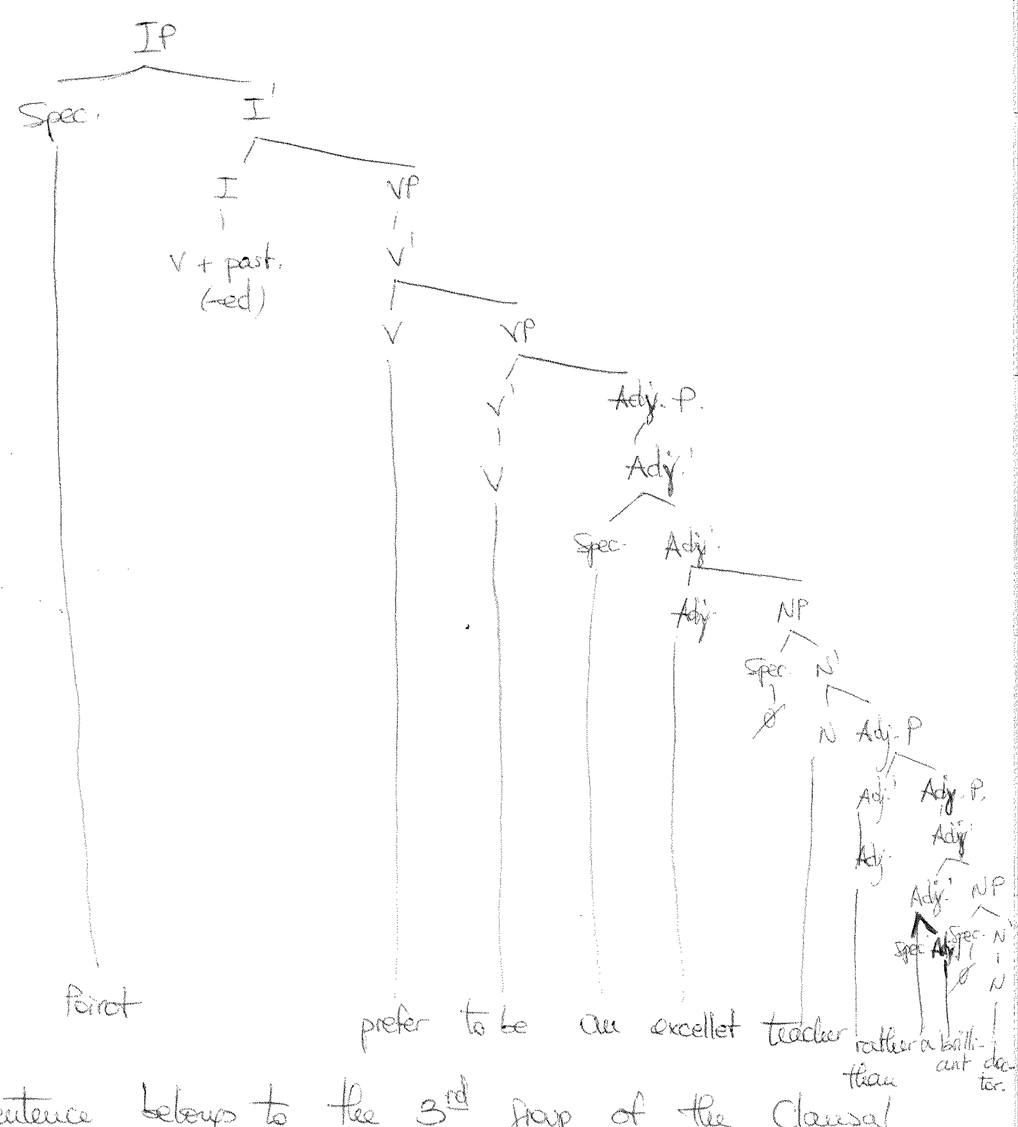

PRO ?

NO THs

This first sentence belongs to the 3rd group of the Clausal Properties, that is caracteristic because of the use of 'prefer'.

b) - Poirot, turned out to be, an excellent teacher, rather than a brilliant doctor.

Subj. (Poirot, N.) — Prop. 1 (turned out to be, V) — D.O. (an excellent teacher; excellent: Adj) — Adjunct (rather than, Superlative) — D.O. / Prop. 2. (a brilliant doctor; brilliant: Adj)

0'25/1

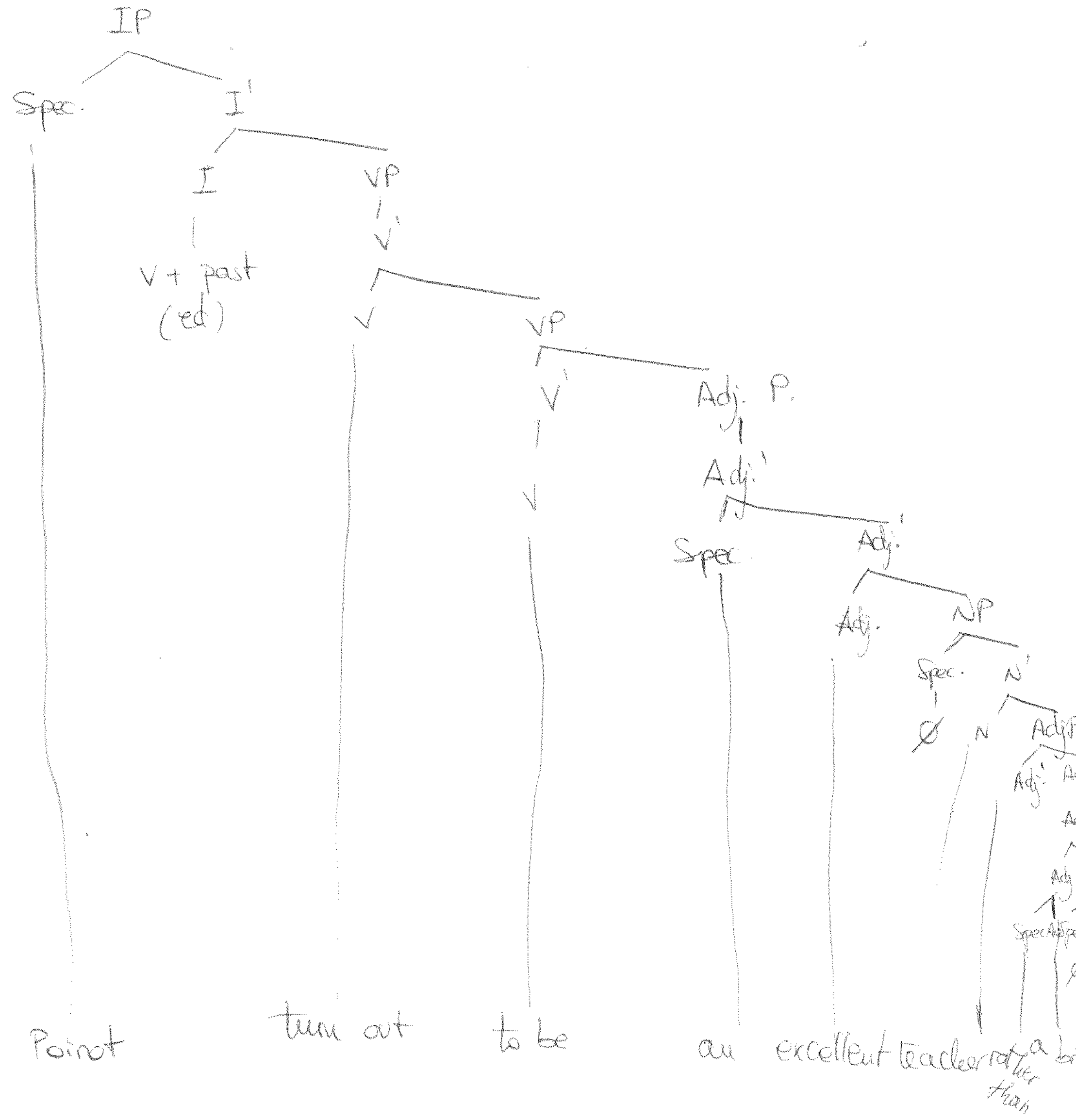

This second sentence also belongs to the Clausal proper-ties. It belongs to the 4th group, because of the verb turned out.

UNIVERSIDAD DE MALAGA
DEPARTAMENTO DE FILOLOGIA INGLESA,
FRANCESA Y ALEMANA

[redacted]

c) – Subj. Poirot | Prop. 1. was liable (V) to be, an excellent (Adj.) teacher | Adjunct: rather than | Prop. 2. a brilliant (Adj.) doctor.

0'2 / 1

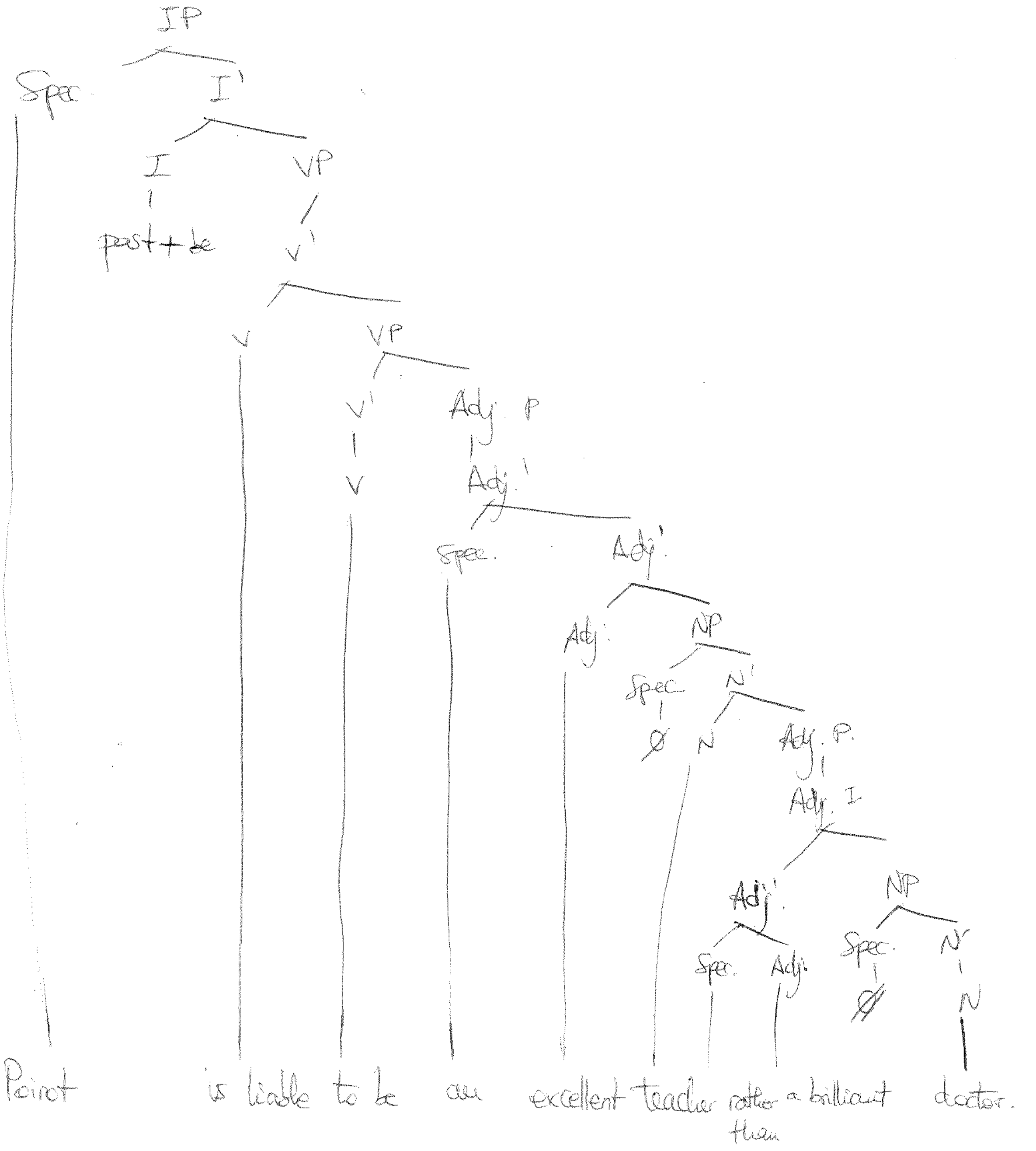

This sentence belongs to the 2nd group of clausal properties because of the use of "be liable". ?

Prop. 1.   Prop. 2

2.) - a) I [subj.] Consider [V] [Pred.] very much him to be [V] a good candidate.

0/1

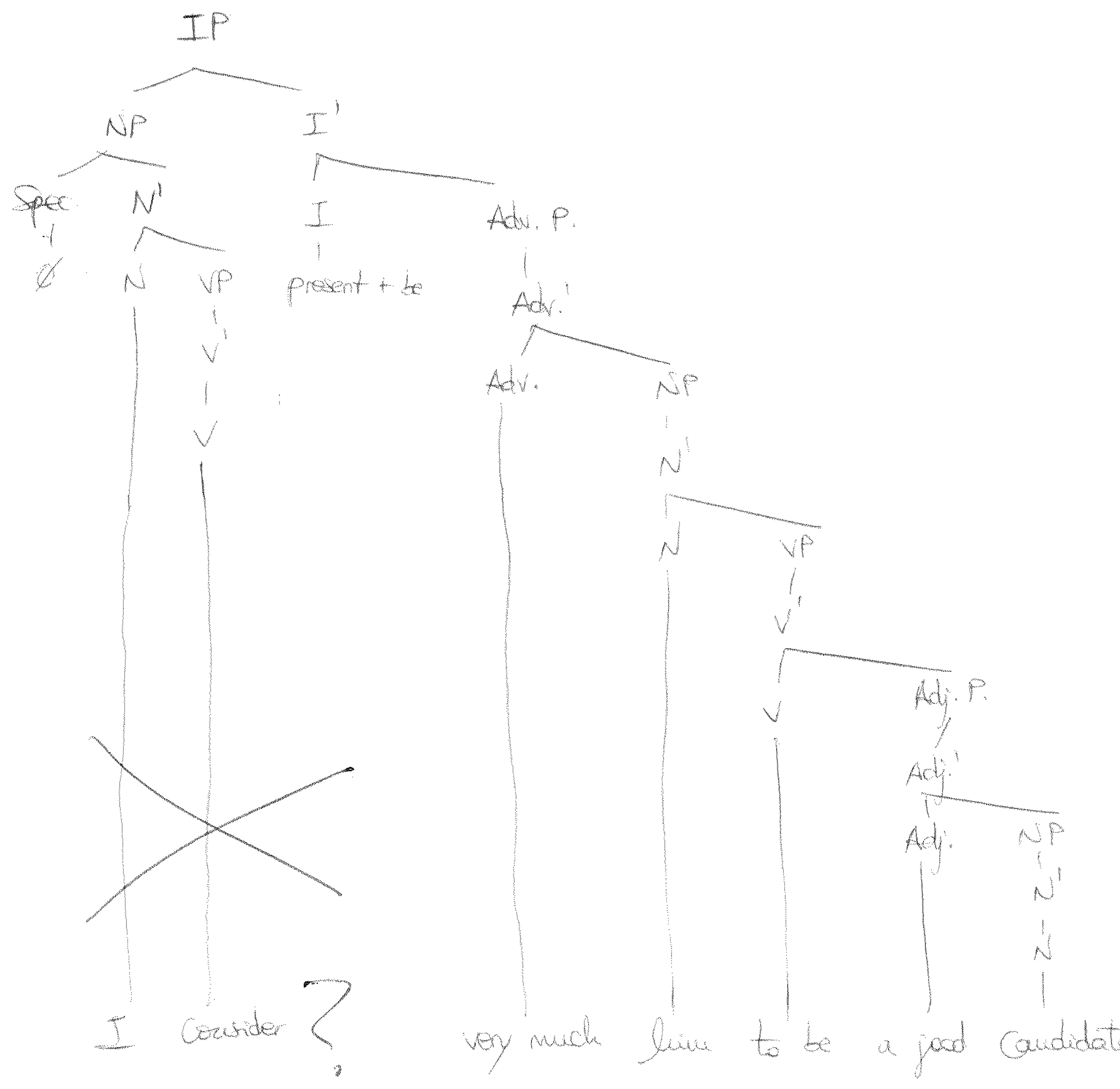

This sentence is ungrammatical.

Why?

UNIVERSIDAD DE MALAGA
DEPARTAMENTO DE FILOLOGIA INGLESA,
FRANCESA Y ALEMANA

[redacted]

b) – Miss Marple (Subj.) surely gave (V) her part to Janvier. (Pred.) (D.O.)

0'2/1

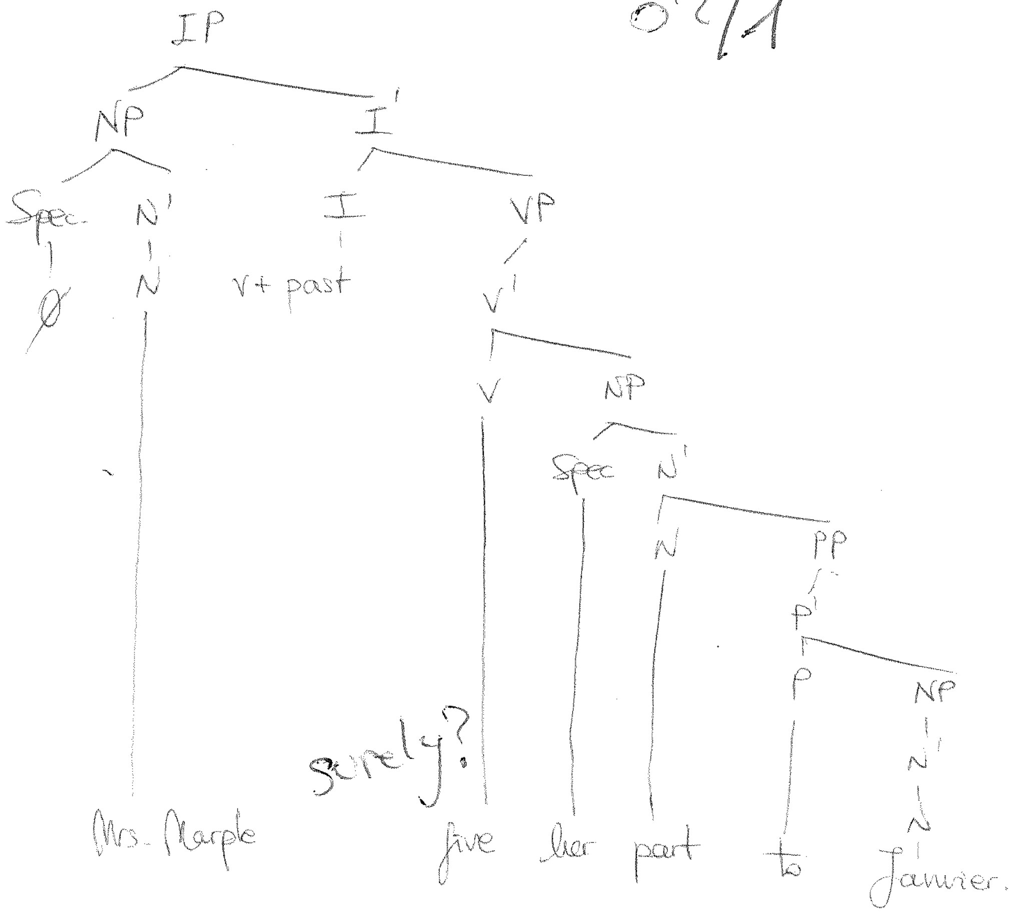

This sentence is grammatical

3.) – For him to have agreed to the proposal is surprising. 0/1

The use of 'is surprising' at the end of the sentence, because with the use of 'For' at the beginning of the sentence it is exceptional to put at the end what makes a reaction to the actor.

ECM?

4.) – a) *Your parents to come to my wedding would be a smart move.

For your parents, to come to my wedding would be a smart move. 0'5/0'5

b) * Mary's concern him.
He concerns Mary. ?

c) * Poirot travelled John and me.
Poirot travilled with John and me. 0'5/0'5

d) * Patrick$_1$ should wash themselves$_1$ every day.
~~Themselves should~~ be washed by Patrick every day.

5.) – a) – This sentence is ungrammatical, because of the use of 'to be'. The correct form would be with 'is'.

b) – I think the second part of the sentence is an IP because it is compound by Subj + V + Complement. 0/1

UNIVERSIDAD DE MALAGA
DEPARTAMENTO DE FILOLOGIA INGLESA,
FRANCESA Y ALEMANA

[redacted]

Fecha [redacted]

0/1

6.) – He was fascinating by everything

This is a sentence written in ~~active~~ passive voice, but the use of 'by everything' makes [we can] confusse it with a passive voice sentence.

SELF

[We can see] that the sentence is an active one because of the order of the elements in the sentence. (Subj. + V + Compl.)

The use of the verb 'was fascinating' is in past continuous because it is an action that it has an impact in the person that is affected by the action. Although it is a past action, in this moment this person is affected by the action.
