# Supplementary material for: Main Ingredients for Success in L2 Academic Writing: Outlining, Drafting and Proofreading
Source: PLoS One. 2015 Jun 5;10(6):e0128309. doi: 10.1371/journal.pone.0128309 (PMC4457904; doi:10.1371/journal.pone.0128309)
Supplement: S1 Appendix — (DOCX) [file pone.0128309.s005.docx]

**Appendix**

1. What do you think about going to University and about being a University student? What could you say about University life from an academic point of view?
2. How important is written language in your degree study? How is your academic writing relevant to your future job?
3. Which expectations did you have on this degree?
4. In the subjects of English linguistics, what is evaluated positively in an academic essay? What do you think is more important in a Linguistics essay: content or expression?
5. Do you enjoy when writing academic essays? Describe your feelings and experiences.
6. Regarding academic writing: what does ‘English academic writing’ mean to you? What do you consider the features of academic writing to be? (Probes: what about the structure of academic texts, what about introducing your opinion in an academic essay, what about punctuation, what about the use of complex sentences and examples?).
7. (I show the interviewee 3 different types of texts in English: personal email paragraph, business letter in English, and academic fragment from a student’s script): is there any difference among these three types of texts? Which one(s) would you consider ‘academic writing’? Can you see any particular features in it?
8. In your degree of English philology: what kind of instruction about academic genre features do you receive? How about the teaching of linking ideas in an academic text?
9. What have you had help with in developing your academic writing?
10. What kind of support would you like to have more of?
11. How could we make academic genre teaching more explicit?
12. How were you taught written academic English? (Probes: grammatical or discursive features?).
13. (I show the interviewee 2 fragments from academic scripts; one contains many instances of academic discourse items, the other does not present any): Can you see any difference between both fragments? Which one do you think is more academic? Why? Which one do you think got higher mark?
14. When facing an expository essay: what are your techniques to plan your writing? Do you write short paragraphs and then read them, or all in a row?
15. What is more difficult for you when writing academically in English: finding ideas, finding correct words, organising ideas?
16. Using Campus Virtual and wikis application: each of them writes a few lines, and others complete. They have to summarise an article.
